# A Photoactivatable Free Mycolic Acid Probe to Investigate Mycobacteria–Host Interactions

**DOI:** 10.1021/acsinfecdis.5c00068

**Published:** 2025-04-14

**Authors:** Kingsley C. Agu, Nicholas Banahene, Carolina Santamaria, Christi Y. Kim, Jessica Cabral, Kyle J. Biegas, Casey Papson, Andrew D. Kruskamp, M. Sloan Siegrist, Benjamin M. Swarts

**Affiliations:** † Department of Chemistry and Biochemistry, Central Michigan University, Mount Pleasant, Michigan 48859, United States; ‡ Biochemistry, Cell, and Molecular Biology Graduate Programs, Central Michigan University, Mount Pleasant, Michigan 48859, United States; § Molecular and Cellular Biology Program, University of Massachusetts, Amherst, Massachusetts 01003, United States; ∥ Department of Microbiology, University of Massachusetts, Amherst, Massachusetts 01003, United States

**Keywords:** Mycobacteria, outer membrane, mycolic acids, immunology, macrophages, photoaffinity, click chemistry, synthesis

## Abstract

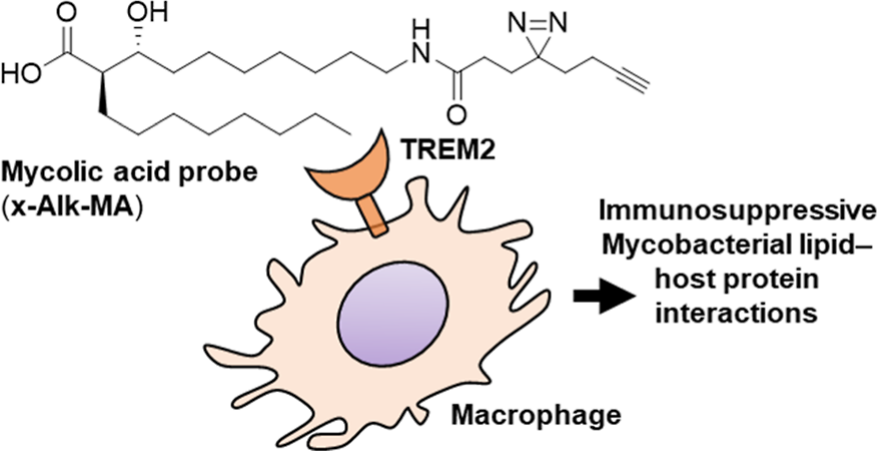

Mycolic acids are long-chain, α-branched, β-hydroxylated fatty acid lipids that populate the outer mycomembrane of mycobacteria, including the pathogen *Mycobacterium tuberculosis*. Mycolic acids predominantly occur in the form of glycolipids, but nonglycosylated free mycolic acids (fMA), which are generated during mycomembrane remodeling, are major constituents of the *M. tuberculosis* biofilm extracellular matrix and promote host immune evasion during *M. tuberculosis* infection. However, our understanding of these processes is nascent, and there is limited information about the fMA–protein interactions involved. To facilitate such studies, we synthesized a fMA analogue probe (x-Alk-MA) containing a photo-cross-linking diazirine and a clickable alkyne to enable live-cell capture and analysis of protein interactors. The synthetic strategy featured asymmetric hydrogenation to establish the β-hydroxy group, diastereoselective alkylation to establish the α-branch, and late-stage modification to install the functional tags. In macrophages, x-Alk-MA recapitulated the cytokine response of native MA and selectively photolabeled TREM2, a host cell receptor for fMAs that suppresses macrophage activation and has been implicated in *M. tuberculosis* immune evasion. The synthetic strategy, chemical probes, and photolabeling methods disclosed herein should facilitate future studies aimed at understanding the roles of fMA in mycobacterial physiology and pathogenesis.

Mycobacteria cause tuberculosis and several related infectious diseases, which are responsible for over 1 million deaths annually.^1^ The success of mycobacteria as pathogens results in part from their complex cell envelope, which is a dynamic structure that is organized into layers of plasma membrane, peptidoglycan, arabinogalactan, and a distinctive outer membrane commonly referred to as the mycomembrane.^2^ The mycomembrane is a highly hydrophobic permeability barrier that contributes to the remarkable tolerance of mycobacteria to stress, including exposure to antibiotics. In addition, mycomembrane components are known to modulate the host immune response to promote bacterial survival during infection. Therefore, the mycomembrane is critical to the physiology and pathogenesis of *Mycobacterium tuberculosis* and related species. Elucidating the molecular details of mycomembrane composition, construction, and host immune interactions may reveal vulnerabilities of these pathogens and provide new avenues for drug and vaccine development.

The mycomembrane primarily consists of large, α-branched, β-hydroxylated fatty acids called mycolic acids, which generally occur as glycoconjugates.^3,4^ Under growth conditions that support replication, mycobacteria enlist the antigen 85 (Ag85) pathway to synthesize arabinogalactan-linked mycolate (AGM), which forms the inner leaflet of the mycomembrane, and trehalose dimycolate (TDM), which is the major constituent of the outer leaflet of the mycomembrane (Figure 1A, left).^5−7^ However, under stress conditions and during infection, the mycomembrane is remodeled through the enzymatic hydrolysis of TDM, which results in the release free mycolic acid (fMA) and trehalose (Figure 1A, right).^8−13^ This stress-induced shift from TDM to nonglycosylated fMA correlates with significant differences in immunological function during infection. TDM acts as a hyper-inflammatory molecule that signals through host receptors, e.g., Mincle (macrophage-inducible C-type lectin), to stimulate pro-inflammatory cytokines and recruit mycobactericidal macrophages.^14−17^ TDM also promotes survival of phagocytosed mycobacteria by inhibiting phagosome maturation and phagosome-lysosome fusion.^18−20^ In contrast, fMA signals through host receptors, e.g., TREM2 (triggering receptor expressed on myeloid cells 2), to recruit mycobacteria-permissive macrophages and promote immune evasion.^21,22^ Thus, one function of mycomembrane remodeling appears to be altering TDM/fMA ratios during infection to enable switching between immunostimulatory and immunosuppressive states.

**Figure 1:**
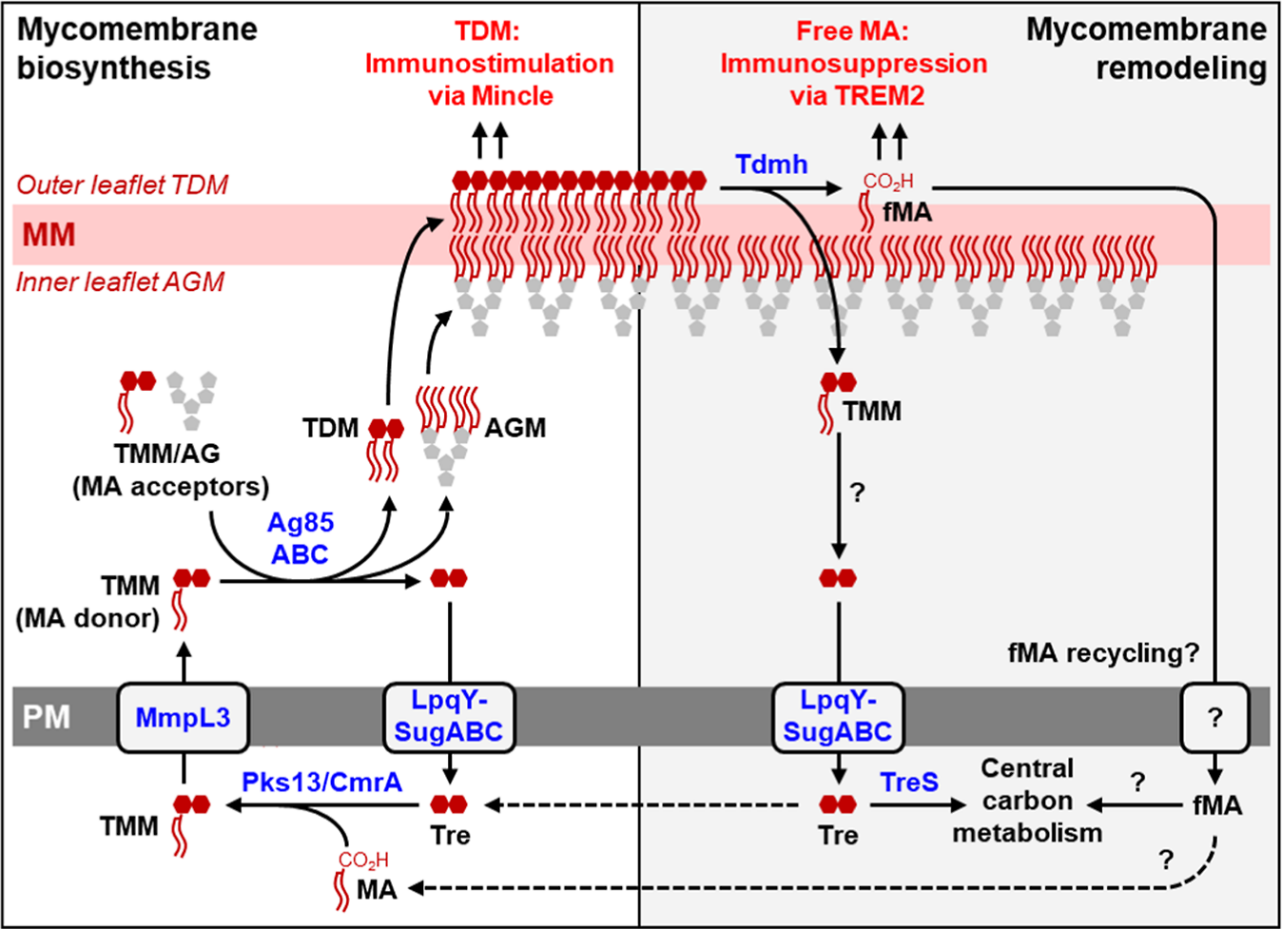
Mycomembrane biosynthesis (left) and remodeling (right) pathways. Mycomembrane remodeling involves breakdown of immunostimulatory TDM to generate immunosuppresive fMA. AG, arabinogalactan; AGM, arabinogalactan-linked mycolate; fMA, free mycolic acid; MM, mycomembrane; PM, plasma membrane; TDM, trehalose dimycolate; TMM, trehalose monomycolate; Tre, trehalose.

In addition to modulating the innate immune response to mycobacterial infection, stress-induced mycomembrane remodeling influences mycobacterial physiology. For example, enzymatic breakdown of TDM to fMA promotes the formation of highly drug-tolerant mycobacterial biofilms containing fMA-rich extracellular matrix.^8,23^ Consistently, nutrient-starved mycobacteria with increased fMA levels have decreased propidium iodide uptake, which is indicative of lower cellular permeability.^13^ Together, these findings suggest that mycomembrane remodeling confers protection to mycobacterial cells. In addition, some evidence suggests that mycobacteria can recycle fMA,^24^ although it has not been determined whether this occurs (or is elevated) during stress, specifically what transporter(s) may be involved, or what the downstream fate of recycled fMA is. Notably, free trehalose that is released through TDM breakdown is known to be recycled via the trehalose-specific transporter LpqY-SugABC^25^ and channeled into central carbon metabolism to help meet energy and antioxidant demands during stress,^12^ and in some cases, back into the mycomembrane for remodeling^13^ (Figure 1A, right). A key player in this trehalose recycling pathway, the trehalose isomerase TreS, has been proposed as a potential target for adjunctive therapeutic development.^12,26^ It is possible that an analogous fMA recycling pathway exists.

The functional consequences and associated mechanisms that result from mycomembrane remodeling-mediated production of fMA, in the context of both bacterium and host, are fundamentally driven by fMA–protein interactions. Generally, such lipid–protein interactions are challenging to discover and characterize, which has motivated the development of chemical probes suited to the task. Bifunctional lipid analogues bearing photo-cross-linking and click chemistry tags have proven particularly useful, as such photoactivatable probes allow (i) covalent labeling of lipid-interacting proteins in live cells and (ii) subsequent proteome-level visualization and identification of the interactors.^27−30^ We previously synthesized a photo-cross-linking and clickable trehalose monomycolate analogue (x-Alk-TMM) and demonstrated its ability to metabolically incorporate into the mycomembrane of the model organism *Mycobacterium smegmatis*, then pulldown and identify mycomembrane proteins on the whole-proteome level.^31,32^ In addition, we recently synthesized a TDM probe variant (x-Alk-TDM) and used it to investigate host protein interactions in macrophages.^33^ In this case, the x-Alk-TDM probe was directly incubated with macrophage cells prior to conducting affinity pulldown and proteomic analysis. We found that x-Alk-TDM interacted with Mincle, the known TDM receptor, as well as intracellular SNARE (soluble *N*-ethylmaleimide-sensitive factor attachment proteins receptor) proteins, a novel finding that provided mechanistic insight into how TDM inhibits phagosome-lysosome fusion to enhance survival of intracellular *M. tuberculosis*.^33^

Here, we expand the toolbox of photoactivatable mycobacterial lipids by synthesizing a photo-cross-linking and clickable fMA probe (x-Alk-MA) and validating its ability to report on fMA–host interactions. We used a stereoselective synthetic strategy to access x-Alk-MA bearing native-like fMA functionalities and carried out macrophage interaction experiments, which revealed that x-Alk-MA elicits an immunosuppressive response similar to that of native fMA, including production of immunosuppressive cytokines and binding to the MA receptor TREM2. Thus, this study confirms the influence of fMA on host cell immune response and, moreover, provides a new way to investigate fMA–protein interactions and the roles they play in mycobacterial physiology and host infection.

## Results and Discussion

### Design of x-Alk-MA Probe

**Figure 2:**
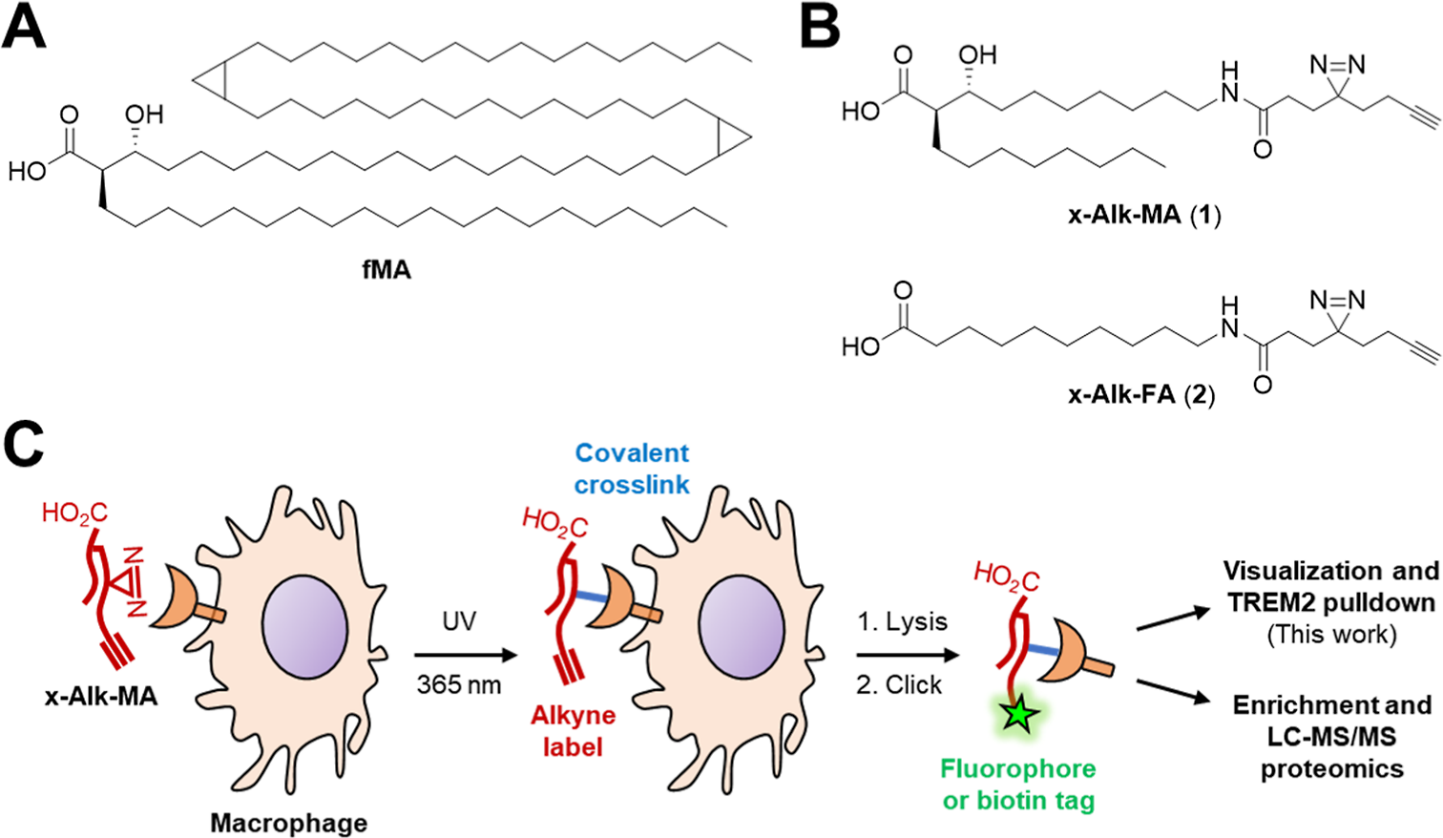
(A) Chemical structure of a representative fMA from *M. tuberculosis*. (B) Photo-cross-linking probes mimicking fMA (x-Alk-MA) and linear fatty acid (x-Alk-FA) developed in this study. (C) Scheme for photolabeling and analysis of x-Alk-MA-interacting host proteins.

We first set out to design an fMA-mimicking photoactivatable probe that is (i) structurally similar to native fMA, (ii) modified with appropriate photo-cross-linking and click groups, and (iii) synthetically accessible. The chemical structure of a representative fMA from *M. tuberculosis* is shown in Figure 2A.^3^ fMAs have a conserved core structure near the polar headgroup, which includes the carboxyl group, an (*R*)-configured branching chain at the α-position, and an (*R*)-configured hydroxy group at the β-position. The chain lengths and additional functionalities on the mero-mycolate chain exhibit significant diversity, differing between species and strains. Depending on the genus and species, the total number of carbon atoms in fMA can range from 22 to 100, and the mero-mycolate chain can be either unmodified or modified with a variety of groups (e.g., alkene, cyclopropyl, keto, methoxy, epoxy).^3^

For our first-generation photoactivatable fMA probe, x-Alk-MA, we included the conserved core region of fMA but opted for relatively short lipid tails that lack organism-specific modifications. Specifically, x-Alk-MA (**1**) maintains the carboxyl headgroup and (*R*)-configured α- and β-substituents of native fMA, but has only 26 total carbons, which is on the small end of the range for fMAs (Figure 2B). We reasoned that a smaller probe design would improve synthetic tractability and avoid solubility problems in photolabeling experiments, while retaining the key immunomodulatory properties being investigated in this study. Although fMA chain length and modifications (e.g., cyclopropanation) can influence activity in certain contexts,^34−37^ signaling through TREM2 can be mediated by a wide variety of lipid ligands,^38,39^ and fMA-mediated TREM2 signaling appears to be largely dependent on the glycosylation state of the headgroup.^21^ Furthermore, our previously published photoactivatable glycolipid probes, x-Alk-TMM and x-Alk-TDM, have shortened lipid chains (15 total carbons per chain) but still maintained on-target labeling of biologically relevant proteins.^31−33^ To enable initial structure–activity relationship (SAR) evaluation, we also designed a linear fatty acid probe, x-Alk-FA (**2**), to test whether the α- and β-functionalities of the fMA core region are necessary for host receptor photolabeling (Figure 2B). Both x-Alk-MA and x-Alk-FA were modified with an amide-linked 8-carbon fragment bearing diazirine and terminal alkyne groups to allow photo-cross-linking and click chemistry-mediated labeling, respectively, which would permit versatile downstream analysis of host protein interactors (Figure 2C).

### Stereoselective Synthesis of x-Alk-MA Probe

**Scheme 1:**
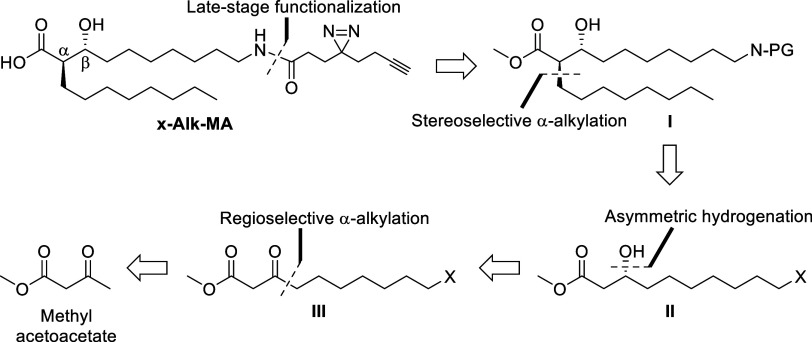
Retrosynthetic Analysis of x-Alk-MA. PG, Protecting Group; X, Halogen Leaving Group

Elegant syntheses of fMA derivatives with the natural (*R*,*R*) stereochemical configuration at the α- and β-positions have previously been accomplished through various approaches,^40^ but the inclusion of chemical tags in these structures adds challenges and has been limited. In 2019, Lesur et al. synthesized a TMM-based metabolic labeling probe bearing a native-like mycolate group with a clickable terminal alkyne on the mero-mycolate chain to enable bacterial cell labeling experiments.^41^ Inspired by this work, we took a similar approach to synthesizing our target molecule, x-Alk-MA. From a retrosynthetic viewpoint (Scheme 1), we envisioned that x-Alk-MA could be accessed through late-stage selective *N*-acylation of an amine-terminated mero-mycolate chain with an appropriate diazirine/alkyne-containing building block, which would avoid exposing these relatively sensitive functional groups to incompatible reaction conditions. To enable this, we designed intermediate **I**, outfitted with a latent amine on the terminus of the mero-mycolate chain. Notably, structure **I** can be considered a versatile synthetic intermediate that could be used to diversify the fMA scaffold with any chemical tag of choice. Establishment of the correct stereochemical relationship in intermediate **I** would be accomplished by diastereoselective α-alkylation of **II** directed by the β-(*R*)-hydroxy group, which itself would be established through (*R*)-enantioselective hydrogenation of ketoester **III**. Intermediate **III** could be synthesized from methyl acetoacetate via regioselective alkylation of the γ-position, simultaneously installing a halogen leaving group X to allow later substitution with a latent amine.

**Scheme 2:**
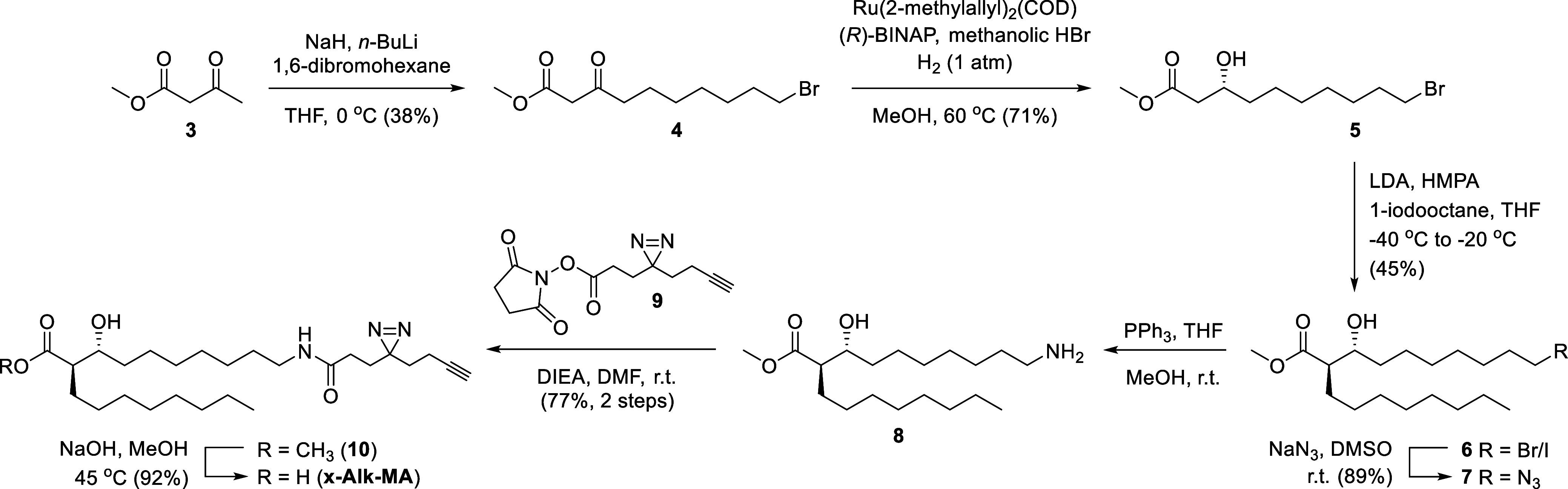
Synthesis of x-Alk-MA (**1**)

The synthesis of x-Alk-MA, shown in Scheme 2, was initiated through regioselective monoalkylation of methyl acetoacetate (**3**) at the γ carbon with 1,6-dibromohexane. Sequential treatment of **3** with NaH and *n*-BuLi generated its corresponding dianion, which was then preferentially S_N_2-alkylated at the more reactive γ-anion^42^ with 1,6-dibromohexane to give compound **4** in 38% yield. ^1^H NMR analysis confirmed that the bromohexyl group was added to the γ-position, as the γ-CH_3_ group of compound **3**, a singlet at 2.2 ppm, was converted to a triplet at 2.5 ppm, representing the newly formed γ-CH_2_ group. This γ-alkylation step served to add the mero-mycolate-mimicking chain containing a terminal bromo group, which was positioned for later S_N_2 reaction to install a latent amine. Next, the (*R*)-β-hydroxy group was established through Noyori asymmetric hydrogenation.^43^ Intermediate **4** was exposed to an in situ-generated chiral catalyst, (*R*)-2,2′-bis(diphenylphosphino)-1,1′-binaphthyl (BINAP)-ruthenium complex, in the presence of atmospheric hydrogen, which converted β-keto ester **4** to the corresponding β-hydroxy ester **5** in 71% yield. NMR evidence of successful reduction included disappearance of the ^13^C NMR signal for the ketone of **4** at 202 ppm and appearance of a signal for the new β-C–OH of **5** at 67.9 ppm; appearance of ^1^H NMR signals for the new β-CH and –OH at 4.0 and 2.9, respectively; and transformation of the α-CH_2_ from a singlet at 3.4 ppm to a pair of doublet-of-doublets around 2.5 ppm, consistent with formation of a chiral center at the β-position.

**Figure 3:**
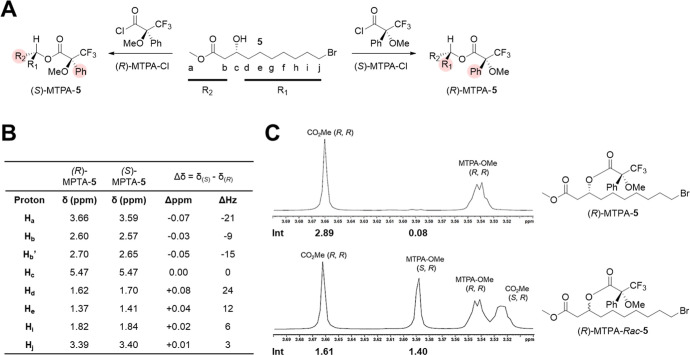
Determination of absolute configuration and enantiomeric ratio of compound **5**. (A) Synthesis of Mosher esters. Shielding of β-substituents by phenyl group in the preferred conformer is indicated by shading. (B) Table of ^1^H NMR chemical shift differences of Mosher esters. (C) Determination of enantiomeric ratio of **5** by NMR integration comparison of Mosher ester of **5** (top) and a racemic mixture of **5** (bottom).

Similar to Lesur et al.,^41^ the absolute stereochemical configuration of β-hydroxy ester **5** was determined through Mosher ester analysis, which involves reaction of the alcohol with chiral derivatizing agents to form diastereomeric esters, followed by ^1^H NMR chemical shift analysis.^44^ The product of asymmetric hydrogenation of **4**, presumed to be structure **5** based on the use of the (*R*)-BINAP ligand, was reacted with either (*R*)-α-methoxy-α-(trifluoromethyl)phenylacetyl chloride (MTPA-Cl) or (*S*)-MTPA-Cl to generate the corresponding Mosher esters, (*S*)-MTPA-**5** and (*R*)-MTPA-**5**, respectively (Figure 3A). Based on the differential shielding effect of the phenyl group on the β-substituents (*R*_1_ and *R*_2_) in the preferred conformations of the diastereomeric esters (Figure 3A), the configuration of the chiral center can be deduced from the difference in chemical shift between the esters. The observed chemical shift differences, shown in Figure 3B, indicate greater phenyl-shielding of R_2_ (methyl ester portion) in (*S*)-MTPA-**5** and greater shielding of *R*_1_ (alkyl chain portion) in (*R*)-MTPA-**5**, respectively, consistent with the expected (*R*) stereochemistry of the β-hydroxy group. Together with employment of the (*R*)-BINAP ligand in the asymmetric hydrogenation, these data unambiguously confirm that the β-stereochemistry of compound **5** matches that observed in natural fMAs. The enantiomeric ratio of compound **5** was determined to be >97% by ^1^H NMR integration analysis of diastereomers present in (*R*)-MTPA-**5** and its racemate (*R*)-MTPA-*Rac*-**5** (Figure 3C), the latter of which was generated through NaBH_4_ reduction of **4** followed by Mosher ester formation.

**Scheme 3:**
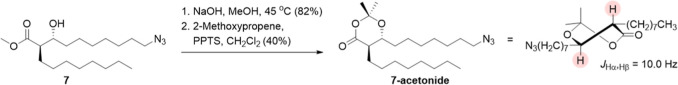
Determination of Stereochemical Configuration of Compound **7** through Synthesis and ^1^H NMR Analysis of Cyclic Derivative **7**-Acetonide

Following successful completion of the enantioselective ketone reduction, the newly formed β-hydroxy group was exploited to direct the subsequent Fráter–Seebach α-alkylation^45,46^ (Scheme 2). Compound **5** was subjected to lithium diisopropylamide (LDA)-mediated α-alkylation with 1-iodooctane in the presence of hexamethylphosphoramide (HMPA) at a temperature maintained between −40 to −20 °C. This step installed the α-branch chain in 45% yield. The product, compound **6**, was isolated as a mixture of the bromo and iodo derivatives, suggesting that nucleophilic iodide released from 1-iodooctane displaced a portion of the bromo group during the alkylation reaction. This mixture was resolved in the subsequent step, as the terminal bromo/iodo groups of mixture **6** were swapped for an azido group by S_N_2 displacement with sodium azide, generating compound **7** as a single diastereomer in 89% yield. At this stage, the stereochemical outcome of the preceding α-alkylation step was assessed by conversion of compound **7** into the corresponding cyclic acetonide derivative and ^1^H NMR coupling constant analysis (Scheme 3). Methyl ester **7** was saponified and reacted with 2-methoxypropene in the presence of pyridinium *p*-toluenesulfonate (PPTS) to generate **7**-acetonide. The *J*_Hα_,_Hβ_ coupling constant of **7**-acetonide was calculated to be 10.0 Hz, confirming the anticipated anti stereochemical relationship between the α-alkyl chain and the β-hydroxy group in compound **7**. Our ^1^H NMR results are consistent with those reported by van der Peet et al.,^47^ who employed the same approach to establish the stereochemical configuration of a structurally similar acetonide derivative.

With the MA core structure established, the remaining steps focused on introduction of the photo-cross-linking and click chemistry groups (Scheme 2). The azido group of compound **7** enables flexible modification through azide-specific click chemistries or azide reduction to an amino group followed by modification with an amine-reactive reagent. In this study, azide reduction was accomplished through Staudinger reaction with triphenylphosphine, yielding intermediate amine **8**, which was taken directly to the next step. As a building block to install the photo-cross-linking diazirine and alkyne tags, we employed *N*-hydroxysuccinimide (NHS) ester **9**, which was prepared according to a reported procedure.^48,49^ Accordingly, amino-MA intermediate **8** was coupled with NHS ester **9**, generating intermediate **10** in 77% yield over two steps from compound **7**. Selective *N*-acylation of **8** by **9** was confirmed by ^1^H NMR analysis, which showed a significant downfield shift of the CH_2_–N absorption (2.67 ppm → 3.16 ppm) but not the β-CH–O absorption (3.69 ppm → 3.62 ppm) on the mero-mycolate chain of compound **10** following the reaction. Finally, methyl ester **10** was subjected to saponification, delivering x-Alk-MA (**1**) in 92% yield.

**Scheme 4:**
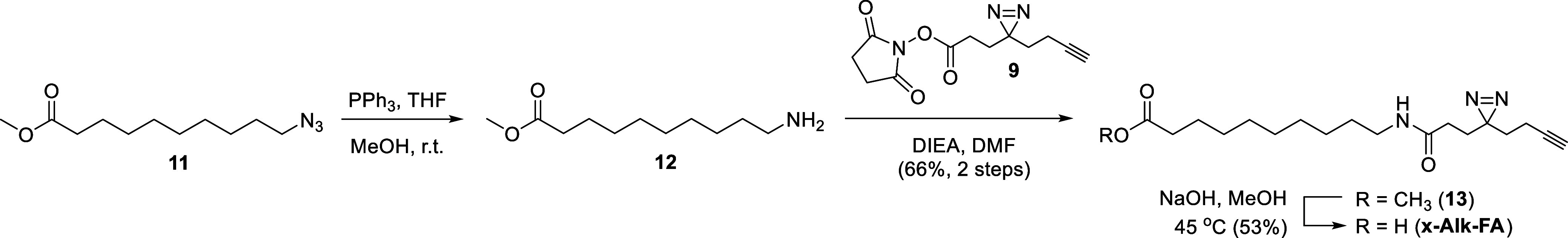
Synthesis of x-Alk-FA

The synthesis of x-Alk-FA (**2**), bearing a linear lipid tail, was prepared through a straightforward route similar to the final stage of x-Alk-MA synthesis (Scheme 4). Known azido ester **11**(50) was subjected to Staudinger reduction to form amine **12**, which was *N*-acylated by NHS ester **9** to give methyl ester **13** in 66% yield over two steps. Saponification of **13** provided x-Alk-FA in 53% yield. As x-Alk-FA has the same mero-mycolate-mimicking chain as x-Alk-MA but lacks α-branch and β-hydroxy groups, it offers a way to begin initial SAR studies, i.e., investigating the contributions of these fMA-specific modifications to biological function.

### x-Alk-MA Probe Recapitulates fMA Activity and Photo-Labels TREM2 in Macrophages

**Figure 4:**
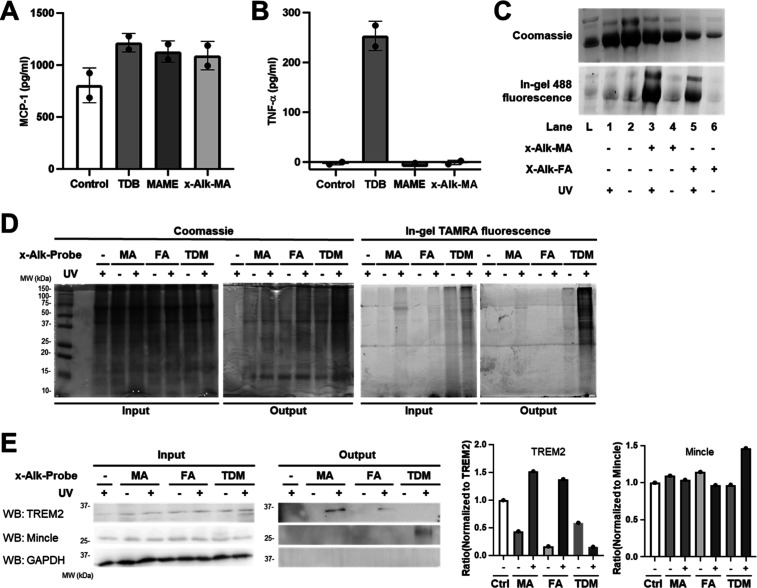
(A,B) ELISA detection of pro-inflammatory cytokines (A) MCP-1 and (B) TNF-α after 24 h incubation of iBMDMs from C57BL/6 mice with x-Alk-MA, TDB, and MAME. Data are from two independent experiments performed in technical triplicate. (C) UV-dependent photo-cross-linking of BSA with x-Alk-MA or x-Alk-FA followed by CuAAC-mediated fluorescence labeling and SDS-PAGE analysis with fluorescence scanning. (D,E) x-Alk-MA, x-Alk-FA, and x-Alk-TDM-mediated affinity enrichment of host interacting proteins. Human THP-1 cells were incubated with x-Alk probes at 100 μM, UV-irradiated, and lysed. Lysates were reacted with TAMRA-biotin-azide (AzTB) via CuAAC and analyzed by Coomassie and in-gel TAMRA fluorescence (input). Clicked samples were incubated with NeutrAvidin agarose beads (output) to evaluate global enrichment of proteins (D) and specific enrichment of the known fMA receptor TREM2 and TDM receptor Mincle (GAPDH, negative control) (E). (D,E) Representative data for 2–3 independent experiments. (E) Semiquantitation of TREM2 and Mincle photolabeling and enrichment by x-Alk probes based on output blots shown in (E).

With the x-Alk-MA probe in hand, we investigated its interactions with host macrophages. We first tested whether x-Alk-MA stimulated a cytokine response similar to that of commercially available unlabeled, native mycolic acid methyl esters (MAME) originating from *M. tuberculosis*, which were used to approximate the cytokine response of native fMA. Given the distinct, opposing immunomodulatory activities of fMA and TDM (Figure 1), we also compared cytokine responses to the TDM-mimicking adjuvant trehalose-6,6-dibehenate (TDB), which induces a macrophage response like native TDM although its lipid chains are simplified.^51^ We measured the production of two cytokines: monocyte chemoattractant protein-1 (MCP-1), which is stimulated by both fMA and TDM/TDB and leads to recruitment of mycobacteria-permissive macrophages; and tumor necrosis factor (TNF-α), which is stimulated by TDM/TDB and leads to granuloma formation and recruitment of mycobactericidal macrophages.^17,21,52,53^ Immortalized bone marrow-derived macrophages (iBMDMs) from C57BL/6 mice were incubated with x-Alk-MA (5 μg/μL) or an equivalent amount of MAME or TDB and cytokine production was assayed (Figure 4A,B). Similar to fMA and MAME, the synthetic probe x-Alk-MA induced MCP-1 production but did not elicit a TNF-α response. In contrast, TDB was found to trigger both MCP-1 and TNF-α production. Together, our results are consistent with the reported immunoactivities of fMA and TDM, and demonstrate that x-Alk-MA, despite its structural modifications, recapitulates the reported immunosuppressive cytokine response of fMA.

Next, we evaluated the ability of x-Alk-MA to photolabel proteins. First, we validated the diazirine and alkyne functionalities of x-Alk-MA in a model photo-cross-linking experiment with purified bovine serum albumin (BSA), essentially as we previously reported.^31^ Briefly, BSA was incubated with x-Alk-MA (or the linear version x-Alk-FA), UV-irradiated, subjected to Cu-catalyzed azide–alkyne cycloaddition (CuAAC) with an azido-488 fluorophore, and analyzed by SDS-PAGE with fluorescence scanning (Figure 4C). Probe-treated and UV-exposed BSA samples showed much higher in-gel fluorescence than negative controls, validating that both x-Alk-MA and x-Alk-FA covalently photo-cross-link proteins and allow subsequent click chemistry-mediated detection.

Finally, we investigated the interaction of x-Alk probes with host macrophage proteins. In addition to x-Alk-MA and x-Alk-FA, we included in the analysis x-Alk-TDM, our reported photoactivatable mimic of TDM bearing linear, truncated chains with diazirine and alkyne groups. The human monocytic leukemia cell line, THP-1, was treated with x-Alk probes, irradiated with UV light, and subjected to CuAAC with TAMRA-biotin-azide (AzTB), a trifunctional reagent that permits downstream enrichment and/or visualization. After affinity enrichment of photolabeled proteins on streptavidin beads and SDS-PAGE analysis, we visualized x-Alk-interacting proteins via in-gel TAMRA fluorescence (Figure 4D). All three x-Alk probes labeled and enriched proteins in a probe- and UV-dependent manner. Interestingly, overall x-Alk-TDM protein labeling was more efficient than that of x-Alk-MA or x-Alk-FA, although the latter two probes did label proteins above background. We next performed immunoblotting to analyze for probe interactions with specific host cell surface receptors, including TREM2, a known receptor for MA, and Mincle, a known receptor for TDM (Figure 4E).^17,21^ Both x-Alk-MA and x-Alk-FA exhibited interactions with TREM2, consistent with the ability of TREM2 to bind a variety of lipid ligands.^38^ However, based on semiquantitation of the output blots, the interaction of TREM2 with x-Alk-MA appeared to be modestly stronger than that of x-Alk-FA, suggesting that the native α-branch and β-hydroxy functionalities of the fMA probe may contribute to TREM2 binding (Figure 4E). Although this result may provide some preliminary insight into TREM2 ligand preference, we acknowledge that immunoblot analysis is not ideal for quantifying binding affinity and more rigorous approaches are necessary to test this further. More clearly, neither x-Alk-MA nor x-Alk-FA detectably interacted with Mincle. By contrast, we observed x-Alk-TDM interactions with Mincle, as we reported before,^33^ but not with TREM2. Notably, none of the x-Alk probes photolabeled an abundant housekeeping protein used as a negative control, glyceraldehyde-3-phosphate dehydrogenase (GAPDH) (Figure 4E). Taken together, these findings demonstrate the ability of the x-Alk-MA probe to elicit a biologically relevant innate immune response and confirm its effectiveness in detecting a known host protein interaction.

## Conclusion

For decades, the virulence-associated, mycolic acid-containing glycoconjugates of the mycomembrane, including TDM, have been a major focus of basic research and drug development for *M. tuberculosis* and related mycobacterial pathogens. However, the occurrence and biological functions of nonglycosylated fMAs have only recently begun to be revealed. One strategy that mycobacteria employ to counteract stress, including host immune pressure, is enzymatic hydrolysis of TDM to generate fMAs, which have been implicated in the formation of drug-tolerant biofilms and host immune evasion. The discovery and mechanistic investigation of such processes presents a challenge, as fMAs are structurally complex and heterogeneous, and they are not directly genetically encoded, which limits the use of traditional experimental approaches. Novel chemical probes, among them photoactivatable analogues, have proven valuable for studying a variety of mycobacterial envelope components, including the trehalose mycolates. Here, we developed the first chemical probe designed specifically to study fMAs. To enable the discovery and characterization of fMA–protein interactions, we synthesized a photoactivatable fMA analogue, x-Alk-MA. Stereoselective transformations and NMR characterization methods were employed to establish and confirm the (*R*,*R*) stereochemical configuration of the α-alkyl and β-hydroxy groups that are present in naturally occurring fMAs. Incorporation of diazirine and terminal alkyne functional groups within the mero-mycolate chain allowed photo-cross-linking and CuAAC-mediated visualization/capture of fMA-interacting proteins in cells. In macrophage experiments, x-Alk-MA bound the host receptor TREM2 and induced an immunosuppressive cytokine response, similar to native fMA. Overall, this study developed and validated x-Alk-MA as a fMA-mimicking photolabeling probe to study mycobacteria–host interactions, and our data help to substantiate a role for fMAs in host immune evasion.

The present study opens several lines of future research. On the probe design level, here we made judicious simplifications to fMA structure to facilitate the synthesis of x-Alk-MA. Although we maintained the core structure including α- and β-functionalities of fMA in the probe, exploring longer chain lengths and other modifications is of interest. In this regard, the developed synthetic strategy provides opportunities to investigate fMA structure–activity relationships. For example, the synthetic route could be adapted to access panels of fMA probes with systematically varied α- and β-functionalities and/or chain lengths, as well as chemical tags, enabling testing of the contributions of these features to fMA function in different contexts. In the present work, this was accomplished to a modest degree by comparing TREM2 binding between x-Alk-MA and the linear, unfunctionalized probe x-Alk-FA. Another aspect to investigate is whether inclusion of a linker amide within the mero-mycolate chain of x-Alk-MA impacts the function relative to fMA. In cellular immune response and protein interaction experiments, the mode of probe administration could be a key factor to explore, as there may be differences between incubating macrophages directly with synthetic probe—as done here—versus exposing macrophages to lipid-coated beads or whole mycobacteria with labeled envelope lipids. With respect to potential avenues of biological inquiry, the availability of x-Alk-MA provides an unprecedented ability to investigate fMA interactions in live bacterial or host cells. Beyond the targeted TREM2 host receptor experiments reported here, x-Alk-MA can be enlisted in chemoproteomic studies, akin to our previous work using x-Alk-TMM and x-Alk-TDM probes.^31,33^ Global chemoproteomic profiling of fMA–protein interactions, when combined with complementary genetic and biochemical approaches, could provide novel mechanistic insights into mycobacterial cell envelope remodeling and lipid transport, biofilm formation, and host immune manipulation, all of which contribute to mycobacterial physiology and pathogenesis.

## Experimental Section

### General Experimental for Synthesis

Materials were obtained from commercial sources without further purification. Anhydrous solvents were obtained either commercially or from an alumina column solvent purification system. All reactions were carried out in oven-dried glassware under inert gas unless otherwise noted. Analytical TLC was performed on glass-backed silica gel 60 Å plates (thickness 250 μm) and detected by UV lamp visualization, phosphomolybdic acid stain, and/or ninhydrin stain. NMR spectra were obtained using a Bruker Avance 500 NMR spectrometer. Coupling constants (*J*) are reported in hertz (Hz) with chemical shifts in ppm (δ) referenced to solvent peaks, with the following splitting abbreviations: s = singlet, bs = broad singlet, d = doublet, dd = doublet of doublets, ddd = doublet of doublets of doublets, dt = doublet of triplets, t = triplet, td = triplet of doublets, m = multiplet, q = quartet. High-resolution electrospray ionization (HR ESI) mass spectra were obtained using an Agilent 6230B LC-ESI-MS TOF instrument.

### Methyl 10-Bromo-3-oxo-decanoate (**4**)

To an oven-dried and argon-flushed Schlenk flask containing anhydrous tetrahydrofuran (THF) (125 mL) stirring under argon at 0 °C was added sodium hydride 60% dispersion in mineral oil (3.6 g, 90 mmol). Methyl acetoacetate (**3**) (8.1 mL, 75 mmol) was added and the reaction was stirred for 20 min at 0 °C, after which 2.5 M *n*-butyllithium in hexanes (30 mL, 75 mmol) was added via cannula. After stirring for 30 min at 0 °C, 1,6-dibromohexane (11.5 mL, 75 mmol) was added to the reaction mixture. The reaction was stirred for another 30 min at 0 °C, after which TLC indicated completion. The reaction was quenched by the slow addition of 2.5 M HCl at 0 °C. The reaction mixture was transferred to a separatory funnel and extracted thrice with ethyl ether. The combined organic layer was washed with brine, dried over Na_2_SO_4_, and filtered. The crude reaction material was concentrated via rotary evaporation and purified by silica gel flash column chromatography (hexanes/ethyl acetate 90:10 → 85:15) to give compound **4** (8.05 g, 38%) as a yellow oil. TLC (hexanes/ethyl acetate 4:1): *R*_f_ = 0.40. ^1^H NMR (500 MHz, CDCl_3_): δ 3.69 (s, 3H), 3.41 (s, 2H), 3.36 (t, *J* = 7.0 Hz, 2H), 2.50 (t, *J* = 7.0 Hz, 2H), 1.80 (pent, *J* = 6.5 Hz, 2H), 1.56 (pent, *J* = 7.0 Hz, 2H), 1.39 (pent, *J* = 7.5 Hz, 2H), 1.31–1.29 (m, 4H). ^13^C NMR (126 MHz, CDCl_3_): δ 202.6, 167.6, 52.3, 49.0, 42.9, 33.9, 32.6, 28.7, 28.4, 27.9, 23.2. HRMS (ESI-TOF) *m*/*z*: [M + Na]^+^ calcd for C_11_H_19_BrO_3_Na, 301.0415; found, 301.0417.

### Methyl 10-Bromo-(*R*)-3-hydroxy-decanoate (**5**)

To an oven-dried and argon-flushed Schlenk flask fitted with a stir bar was added (*R*)-BINAP (223 mg, 0.36 mmol) and Ru(2-methyllallyl)_2_(COD) (114 mg, 0.36 mmol); weighing and addition to the flask were done in a glovebox. Anhydrous acetone (14 mL) and 0.176 M methanolic hydrogen bromide solution (3.7 mL, 0.65 mmol) were cannulated into the reaction flask, and the reaction was stirred under argon for 30 min at room temperature. The solvent was thoroughly evaporated under a vacuum to obtain the catalyst as a yellow solid that was used immediately. After backfilling the flask with argon, compound **4** (5.00 g, 17.8 mmol) dissolved in anhydrous methanol (30.0 mL) was added to the catalyst via cannula. The argon atmosphere was replaced with hydrogen (1 atm), and the reaction was stirred at 60 °C for 20 h, after which TLC indicated that all of the starting material was consumed. The reaction mixture was concentrated via rotary evaporation and purified by silica gel flash column chromatography (hexanes/ethyl acetate 9:1 → 4:1) to give compound **5** (3.6 g, 71%) as a clear yellow oil. TLC (hexanes/ethyl acetate 4:1): *R*_f_ = 0.21. ^1^H NMR (500 MHz, CDCl_3_): δ 4.00 (bs, 1H), 3.71 (s, 3H), 3.40 (t, *J* = 6.5 Hz, 2H), 2.91 (d, *J* = 4.5 Hz, 1H), 2.51 (dd, *J* = 5.5, 27.5 Hz, 1H), 2.42 (dd, *J* = 14.5, 27.5 Hz, 1H), 1.84 (pent, *J* = 7.0 Hz, 2H), 1.53–1.26 (m, 10H). ^13^C NMR (126 MHz, CDCl_3_): δ 173.4, 67.9, 51.7, 41.2, 36.5, 34.0, 32.8, 29.3, 28.7, 28.1, 25.4. HRMS (ESI-TOF) *m*/*z*: [M + Na]^+^ calcd for C_11_H_21_BrO_3_Na, 303.0572; found, 303.0564.

### Synthesis and ^1^H NMR Analysis of Mosher Esters of Compound **5**

To a solution of compound **5** or its racemate (18 mg, 0.064 mmol) in anhydrous dichloromethane (1 mL) and anhydrous pyridine (16 μL, 0.20 mmol) stirring at room temperature, (*S*)- or (*R*)-MTPA-Cl (23 μL, 0.12 mmol) was added. The reaction was stirred for 30 min at room temperature, after which TLC indicated completion. The reaction mixture was quenched by addition of water, extracted thrice with ethyl ether, and the combined organic layer was dried over Na_2_SO_4_, filtered, and concentrated via rotary evaporation. The crude reaction material was purified by silica gel flash column chromatography (hexanes/ethyl acetate 4:1) to give MPTA-**5** compounds ((*R*)-MPTA-**5**, 23 mg, 76%) as colorless solids. TLC (hexanes/ethyl acetate 4:1): *R*_f_ = 0.40. ^1^H NMR of (*R*)-MTPA-**5** (300 MHz, CDCl_3_): δ 7.54 (d, *J* = 6.0 Hz, 1H), 7.53 (d, *J* = 7.5 Hz, 1H), 7.41–7.37 (m, 3H), 5.51–5.43 (m, 1H), 3.66 (s, 3H), 3.55–3.53 (m, 3H), 3.39 (t, *J* = 6.6 Hz, 2H), 2.70 (dd, *J* = 7.8, 16 Hz, 1H), 2.60 (dd, *J* = 4.8, 16 Hz, 1H), 1.82 (pent, *J* = 6.6 Hz, 2H), 1.68–1.58 (m, 2H), 1.41–1.31 (m, 2H), 1.28–1.16 (m, 6H). ^1^H NMR of (*S*)-MTPA-**5** (300 MHz, CDCl_3_): δ 7.53 (d, *J* = 5.7 Hz, 1H), 7.52 (d, *J* = 7.5 Hz, 1H), 7.42–7.37 (m, 3H), 5.51–5.43 (m, 1H), 3.59 (s, 3H), 3.55–3.53 (m, 3H), 3.40 (t, *J* = 6.6 Hz, 2H), 2.65 (dd, *J* = 7.8, 16 Hz, 1H), 2.57 (dd, *J* = 5.1, 16 Hz, 1H), 1.84 (pent, *J* = 6.6 Hz, 2H), 1.77–1.63 (m, 2H), 1.46–1.36 (m, 2H) 1.35–1.26 (m, 6H). ^1^H NMR of (*R*)-MTPA-*Rac*-**5** (300 MHz, CDCl_3_): 7.55–7.50 (m, 4H), 7.43–7.37 (6H), 5.51–5.42 (m, 2H), 3.66 (s, 3H), 3.59 (s, 3H), 3.55–3.53 (m, 3H), 3.53–3.51 (m, 3H), 3.39 (t, *J* = 6.3 Hz, 2H), 3.38 (t, *J* = 6.6 Hz, 2H), 2.74–2.54 (m, 4H), 1.82 (pent, *J* = 6.3 Hz, 4H), 1.74–1.59 (m, 4H), 1.46–1.14 (m, 16H).

### Methyl 10-Bromo-(*R*)-3-hydroxyl-(*R*)-2-octyl Decanoate (**6**)

To an oven-dried and argon-flushed round-bottom flask containing anhydrous THF (20 mL) stirring under argon at −78 °C was added 2.0 M lithium diisopropylamide (LDA) in THF/heptane/ethylbenzene (20.9 mL, 41.9 mmol) via cannula, followed by the addition of a solution of compound **5** (3.91 g, 13.9 mmol) in anhydrous THF (20 mL). The reaction mixture was stirred under argon at −78 °C for 1 h, after which hexamethylphosphoramide (HMPA) (7.3 mL, 41.9 mmol) and a solution of 1-iodooctane (7.60 mL, 41.9 mmol) in THF (30 mL) were added sequentially. The reaction mixture was stirred and allowed to slowly warm to −50 °C and stirred at this temperature for 1 h. The reaction mixture was then stirred for 6 h at a temperature maintained between −40 °C to −20 °C. Saturated ammonium chloride was added to the reaction followed by the addition of water and extraction with ethyl ether three times. The combined organic layer was washed with brine, dried over MgSO_4_, and filtered. The crude material was concentrated via rotary evaporation and purified by silica gel flash column chromatography (hexanes/ethyl acetate 98:2 → 85:15) to give compound **6** (2.43 g, 45%) as a mixture of bromo and iodo derivatives, clear yellow oil. TLC (hexanes/ethyl acetate 4:1): *R*_f_ = 0.50. ^1^H NMR (500 MHz, CDCl_3_): δ 3.71 (s, 1H), 3.68–3.63 (m, 3H), 3.40 (t, *J* = 7.00 Hz, 1H), 3.18 (t, *J* = 7.00 Hz, 1H), 2.45 (m, 1H), 2.43 (m, 1 –OH), 1.88–1.79 (m, 2H), 1.73–1.65 (m, 1H), 1.62–1.57 (m, 1H), 1.48–1.36 (m, 24H). ^13^C NMR (126 MHz, CDCl_3_): δ 176.2, 72.2, 51.6, 51.0, 35.6, 32.8, 31.8, 29.7, 29.5, 29.4, 29.32, 29.30, 29.2, 28.7, 28.1, 27.4, 25.6, 22.7, 14.1. HRMS (ESI-TOF) *m*/*z*: [M + Na]^+^ calcd for C_19_H_37_BrO_3_Na, 415.1824; found, 415.1823. HRMS (ESI-TOF) *m*/*z*: [M + Na]^+^ calcd for C_19_H_37_IO_3_Na, 463.1685; found, 463.1679.

### Methyl 10-Azido-(*R*)-3-hydroxyl-(*R*)-2-octyl Decanoate (**7**)

To an oven-dried round-bottom flask containing anhydrous dimethyl sulfoxide (DMSO) (10.0 mL) stirring under argon was added compound **6** (678.3 mg, 1.730 mmol) and sodium azide (1.12 g, 17.2 mmol). The reaction mixture was stirred at room temperature overnight. The crude reaction mixture was filtered, concentrated via rotary evaporation, and purified by silica gel flash column chromatography (hexanes/ethyl acetate 4:1) to give compound **7** (545.6 mg, 89%) as a yellow oil. TLC analysis (hexanes/ethyl acetate 4:1): *R*_f_ = 0.45. ^1^H NMR (500 MHz, CDCl_3_): δ 3.71 (s, 3H), 3.65 (bs, 1H), 3.25 (t, *J* = 6.5 Hz, 2H), 2.49–2.41 (m, 2H), 1.74–1.67 (m, 1H), 1.62–1.57 (m, 3H), 1.48–1.25 (m, 22H), 0.88 (t, *J* = 6.5 Hz, 3H). ^13^C NMR (126 MHz, CDCl_3_): δ 176.2, 72.2, 51.6, 51.5, 51.0, 35.7, 31.8, 29.7, 29.5, 29.4, 29.2, 29.1, 28.8, 27.4, 26.6, 25.6, 22.7, 14.1.

### Compound **7**-Acetonide

To a solution of compound **7** (526 mg, 1.48 mmol) in methanol (2.5 mL) stirring under argon in a round-bottom flask was added 10% aqueous NaOH (2.5 mL). The reaction was heated to 45 °C and stirred overnight, after which TLC indicated that the starting material was consumed. The reaction was cooled to room temperature, acidified with 1 M HCl, and extracted thrice with ethyl acetate. The combined organic layer was dried over MgSO_4_ and filtered. The crude reaction material was concentrated via rotary evaporation and purified by silica gel flash column chromatography (hexanes/ethyl acetate 1:1) to give the carboxylic acid intermediate (412 mg, 82%) as a pale-yellow oil. A portion of the intermediate (42 mg, 0.12 mmol) was dissolved in anhydrous CH_2_Cl_2_ (3 mL) and stirred under argon at room temperature. To the solution was added 2-methoxypropene (40 μL, 0.42 mmol) and pyridinium *p*-toluene sulfonate (PPTS) (2.3 mg, 0.0092 mmol). The reaction mixture was stirred for 1 h at room temperature, after which NaHCO_3_ (9.0 mg, 0.11 mmol) was added. The reaction mixture was stirred for 5 min at room temperature and concentrated via rotary evaporation. The crude product was purified by silica gel flash column chromatography (hexanes/ethyl acetate 10:1 containing 1% Et_3_N) to give product **7**-acetonide (18.5 mg, 40%) as a pale yellow syrup. TLC analysis (hexanes/ethyl acetate 9:1 containing 1% Et_3_N): *R*_f_ = 0.40. ^1^H NMR (500 MHz, CDCl_3_): δ 3.90 (dt, *J* = 1.5, 10.0 Hz, 1H), 3.26 (t, *J* = 7.0 Hz, 2H), 2.32 (dt, *J* = 5.0, 10.0 Hz, 1H), 1.84–1.77 (m, 1H), 1.65–1.52 (m, 4H), 1.56 (s, 3H), 1.55 (s, 3H), 1.49–1.22 (m, 21H), 0.88 (t, *J* = 6.5 Hz, 3H). ^13^C NMR (125 MHz, CDCl_3_): δ 171.1, 105.4, 71.0, 51.6, 45.6, 33.9, 32.0, 30.1, 29.5, 29.40, 29.38, 29.2, 29.0, 27.5, 26.8, 25.4, 25.2, 22.8, 14.2.

### Methyl *N*-(3-(3-(But-3-yn-1-yl)-3*H*-diazirin-3-yl)-propanoyl)-10-amino-(*R*)-3-hydroxyl-(*R*)-2-octyl Decanoate (**10**)

To an oven-dried round-bottom flask containing THF (3.0 mL) and methanol (1.0 mL) stirring under argon was added a solution of compound **7** (68.5 mg, 0.192 mmol) in methanol (0.5 mL) followed by triphenylphosphine (101 mg, 0.385 mmol). After stirring for 30 min, water (0.4 mL) was added and the reaction mixture was stirred at room temperature for 20 h, at which point TLC indicated that the starting material was consumed, and a ninhydrin stain-positive spot was observed. The crude reaction material containing compound **8** was concentrated via rotary evaporation and taken directly to the next step without further purification. To a solution of the crude material containing compound **8** in *N*,*N*-dimethylformamide (DMF) (5 mL) in a round-bottom flask stirring under argon was added *N*,*N*′-diisopropylethylamine (DIEA) (100 μL, 0.577 mmol) followed by NHS ester **9**(48,49) (61.9 mg, 0.235 mmol). The reaction mixture was stirred under argon in the dark at room temperature for 20 h. The crude reaction material was transferred to a separatory funnel, diluted with brine, and extracted thrice with ethyl ether. The combined organic layer was dried over MgSO_4_ and filtered. The crude material was concentrated via rotary evaporation and purified by silica gel flash column chromatography (hexanes/ethyl acetate 2:1 → 1:1) to give compound **10** (70.3 mg, 77% over two steps) as yellow-brown waxy solid. TLC (hexanes/ethyl acetate 1:1): *R*_f_ = 0.28. ^1^H NMR (500 MHz, CDCl_3_): δ 5.57 (bs, 1H), 3.68 (s, 3H), 3.62 (bs, 1H), 3.19 (q, *J* = 6.5 Hz, 2H), 2.51 (bs, 1H), 2.41–2.38 (m, 1H), 2.01–1.95 (m, 3H), 1.89 (t, *J* = 7.0 Hz, 2H), 1.81 (t, *J* = 7.5 Hz, 2H), 1.69–1.64 (m, 1H), 1.62 (t, *J* = 7.5 Hz, 2H), 1.59–1.51 (m, 1H), 1.33–1.17 (m, 20H), 0.85 (t, *J* = 8.5 Hz, 3H). ^13^C NMR (125 MHz, CDCl_3_): δ 176.2, 171.0, 82.7, 72.3, 69.2, 51.5, 51.0, 39.6, 35.6, 32.4, 31.8, 30.4, 29.6, 29.52, 29.48, 29.4, 29.2, 29.1, 28.4, 27.9, 27.4, 26.8, 25.6, 22.6, 14.1, 13.3. HRMS (ESI-TOF) *m*/*z*: [M + Na]^+^ calcd for C_27_H_47_N_3_O_4_Na, 500.3464; found, 500.3458.

### *N*-(3-(3-(But-3-yn-1-yl)-3*H*-diazirin-3-yl)-propanoyl)-10-amino-(*R*)-3-hydroxyl-(*R*)-2-octyl Decanoic Acid (x-Alk-MA, **1**)

To a solution of compound **10** (10.0 mg, 0.021 mmol) in methanol (3.0 mL) stirring under argon in a round-bottom flask was added 10% aqueous NaOH (0.45 mL). The reaction was heated to 45 °C and stirred in the dark for 24 h, after which TLC indicated that the starting material was consumed. The reaction was cooled to room temperature, acidified with 1 M HCl, and extracted thrice with ethyl ether. The combined organic layer was dried over MgSO_4_ and filtered. The crude reaction material was concentrated via rotary evaporation and purified by silica gel flash column chromatography (hexanes/ethyl acetate 4:1 → 1:1 → dichloromethane/methanol 6.5:1) to give compound **1** (x-Alk-MA) (9.0 mg, 92%) as a pale-yellow oil. TLC (hexanes/ethyl acetate 1:1): *R*_f_ = 0.05. ^1^H NMR (500 MHz, CD_3_OD): δ 7.98 (s, 1H), 3.70–3.66 (m, 1H), 3.16 (t, *J* = 6.0 Hz, 2H), 2.41–2.36 (m, 1H), 2.28 (t, *J* = 2.5 Hz, 1H), 2.05–2.01 (m, 4H), 1.76 (t, *J* = 7.8 Hz, 2H), 1.63 (t, *J* = 7.5 Hz, 2H), 1.60–1.46 (m, 6H), 1.45–1.25 (m, 22H), 0.92 (t, *J* = 6.8 Hz, 3H). ^13^C NMR (125 MHz): δ 177.3, 172.9, 82.2, 71.9, 68.9, 52.4, 39.1, 34.2, 31.9, 31.6, 29.7, 29.3, 29.18, 29.15, 28.96, 28.92, 28.6, 28.4, 27.5, 27.3, 26.5, 25.2, 22.3, 13.0, 12.4. HRMS (ESI-TOF) *m*/*z*: [M + Na]^+^ calcd for C_26_H_45_N_3_O_4_Na, 486.3308; found, 486.3292.

### *N*-(3-(3-(But-3-yn-1-yl)-3*H*-diazirin-3-yl)-propanoyl)-10-amino-decanoic Acid (x-Alk-FA, **2**)

To an oven-dried round-bottom flask containing THF (1.8 mL) and methanol (0.5 mL) stirring under argon was added a solution of compound **11**(50) (13.5 mg, 0.0594 mmol) in methanol (0.5 mL) followed by triphenylphosphine (31.2 mg, 0.119 mmol). After stirring for 30 min, water (0.5 mL) was added and the reaction mixture was stirred at room temperature for 36 h, at which point TLC indicated that the starting material was consumed, and a ninhydrin stain-positive spot was observed. The crude reaction material containing compound **12** was concentrated via rotary evaporation and taken directly to the next step without further purification. To a solution of the crude material containing compound **12** in DMF (5 mL) in a round-bottom flask stirring under argon was added DIEA (38 μL, 0.22 mmol) followed by NHS ester **9** (11.9 mg, 0.0452 mmol). The reaction mixture was stirred under argon in the dark at room temperature for 20 h, at which point the reaction was stopped. The crude reaction material was transferred to a separatory funnel, diluted with brine, and extracted with ethyl ether. The combined organic layer was dried over MgSO_4_ and filtered. The crude material was concentrated via rotary evaporation and purified by silica gel flash column chromatography (hexanes/ethyl acetate 3:1 → 1.5:1) to give compound **13** (10.4 mg, 66% over two steps) as yellow-brown waxy solid, which was taken to the next step. To a solution of compound **13** (10.4 mg, 0.0298 mmol) in methanol (2.0 mL) stirring under argon in a round-bottom flask was added 10% aqueous NaOH (0.12 mL). The reaction was heated to 45 °C and stirred in the dark for 20 h, after which TLC indicated that the starting material was consumed. The reaction was cooled to room temperature, acidified with 1 M HCl, and extracted with ethyl ether. The combined organic layer was dried over MgSO_4_ and filtered. The crude reaction material was concentrated via rotary evaporation and purified by silica gel flash column chromatography (hexanes/ethyl acetate 4:1 → 1:1, with 1% acetic acid) to give compound **2** (x-Alk-FA) (5.3 mg, 53%) as a pale-yellow oil. TLC (hexanes/ethyl acetate 1:1, with 1% acetic acid): *R*_f_ = 0.28. ^1^H NMR (500 MHz, CD_3_OD): δ 7.95 (s, 1H), 3.13 (t, *J* = 7.0 Hz, 2H), 2.29–2.25 (m, 3H), 2.03–1.99 (m, 4H), 1.73 (t, *J* = 8.0 Hz, 2H), 1.61 (t, *J* = 7.5 Hz, 2H), 1.62–57 (m, 2H), 1.52–1.47 (m, 2H), 1.37–1.28 (10H). ^13^C NMR (125 MHz): δ 177.7, 174.2, 83.6, 70.3, 40.5, 34.9, 33.4, 31.1, 31.0, 30.5, 30.3, 30.2, 29.9, 28.9, 27.9, 26.1, 13.8. HRMS (ESI-TOF) *m*/*z*: [M + Na]^+^ calcd for C_18_H_29_N_3_O_3_Na, 358.2107; found, 358.2100.

### BSA Photo-Cross-Linking and Analysis

The labeling procedure was performed on defatted, reduced, and alkylated bovine serum albumin (BSA) prepared as in previously reported procedures.^31^ Briefly, BSA was incubated with excess CHCl_3_ overnight with stirring. After 12 h, BSA was collected by filtration and dried under air. A 10 mg/mL stock solution in water was prepared and diluted to 3.8 mg/mL. Dithiothreitol (DTT) (100 mM stock in 50 mM ammonium bicarbonate solution) was added to a final concentration of 5 mM and the protein was incubated at 56 °C for 20 min in a thermomixer. To reduced BSA, iodoacetamide was added to a final concentration of 16.5 mM (from 550 mM iodoacetamide stock in 50 mM ammonium bicarbonate solution) and incubated at room temperature in the dark for 20 min. To 90 μL aliquots of reduced and alkylated BSA was added 10 μL of x-Alk-MA or x-Alk-FA to a final probe concentration of 100 μM, BSA concentration of 2.7 mg/mL, DMSO concentration of 10%. The mixture was incubated at room temperature with mixing for 1 h. The BSA-probe mixtures were split into two equal volumes in 1.5 mL centrifuge tubes. The samples were either exposed to UV irradiation for 15 min or not using a 15 W 365 nm UV bench lamp (UVP) at a distance of ∼ 2 cm. All six samples were subjected to Cu-catalyze azide–alkyne cycloaddition (CuAAC) using carboxyrhodamine 110 azide (Click Chemistry Tools) (30 μM) in the presence of sodium ascorbate (1.2 mM), copper(II) sulfate (1 mM) and tris(benzyltriazolylmethyl)amine (TBTA) ligand (128 μM), giving a final BSA concentration of 1.95 mg/mL. The reactions were incubated for 1 h at room temperature. Excess CuAAC reagents were removed by sequential addition of 4 volumes of methanol, 1 volume of chloroform, and 3 volumes of water. Samples were briefly vortexed and centrifuged at 18,000*g* for 5 min. The top methanol layer was carefully aspirated to not to disturb protein wafer. Three volumes of methanol were added, followed by brief vortex and centrifugation at 18,000*g* for 5 min followed by careful aspiration. The resulting protein pellets were dried for 10 min and resuspended in 100 μL water. Twenty μg of each sample was analyzed by 4–20% SDS-polyacrylamide gel electrophoresis (BioRad) in a Tris-glycine-SDS running buffer, followed by fluorescence scanning using a Typhoon FLA 7000 (GE Healthcare Life Science) with a 485 excitation and 520 emission filter. The gel was fixed for 15 min (40% ethanol, 10% acetic acid in Milli-Q water), rinsed once with Milli-Q water and stained overnight with gentle agitation in QC Colloidal Coomassie stain (Bio-Rad). The gel was rinsed with Milli-Q water until the background was clear and imaged using a ChemiDoc Touch Imaging System (Bio-Rad) and processed by Image Lab software (Bio-Rad).

### Cell Lines and Media

Immortalized bone marrow-derived macrophages (iBMDMs) from C57BL/6J mice were generously provided by Dr. Christopher Sassetti (University of Massachusetts Medical School). The human monocyte/macrophage THP-1 cell line was obtained from Ubigene Biosciences. iBMDMs were cultured in Dulbecco’s Modified Eagle Medium (DMEM; Genesee Scientific) supplemented with 10% (v/v) heat-inactivated fetal bovine serum (FBS; Fisher Scientific), 2 mM l-glutamine, and 10 mM HEPES, and maintained in a humidified incubator at 37 °C with 5% CO_2_ in a humidified incubator. THP-1 cells were cultured in suspension in RPMI 1640 medium (Genesee Scientific) supplemented with 10% (v/v) heat-inactivated FBS (Fisher Scientific), 1% antibiotic-antimycotic (penicillin G, streptomycin, and amphotericin B), 1 mM sodium pyruvate, and 10 mM HEPES (all from Genesee Scientific). Differentiation of THP-1 cells into macrophages was achieved by treating them with 150 nM phorbol-12-myristate 13-acetate (PMA; Sigma) for 48 h.

### Cytokine Production

Immortalized BMDMs were incubated with 5 μg/μL of x-Alk-MA, purified mycolic acid methyl esters (MAME) (BEI Resources, Cat. no. NR-14854) or trehalose-6,6-dibehenate (TDB; InvivoGen) for 24 h. Supernatants were collected at 24 h and TNF-α and MCP-1 production were measured by enzyme-linked immunosorbent assay according to kit instructions (R&D Systems, DuoSet ELISA).

### Macrophage Protein Photo-Cross-Linking and Analysis

Stock solutions of x-Alk-MA, x-Alk-TDM, and x-Alk-FA were prepared in anhydrous DMSO (100 mM) and stored at −20 °C. Additional reagents used for copper-catalyzed alkyne–azide cycloaddition (CuAAC) included sodium ascorbate (60 mM in H_2_O, freshly prepared, Sigma), TBTA (6.4 mM in DMSO; Click Chemistry Tools, stored at −20 °C), CuSO_4_ (50 mM in H_2_O, Sigma, stored at room temperature (RT)), and TAMRA biotin azide (AzTB, 10 mM in DMSO; Click Chemistry Tools, stored at −20 °C). Differentiated THP-1 cells (5 × 10^6^ cells/plate) were incubated ± probes (100 μM in DMSO) for 6 h, UV-irradiated (15 W, 365 nm UV lamp, 10 min, ice), washed with PBS, and lysed in RIPA buffer (1 mM PMSF, 1× complete EDTA-free protease inhibitor; Thermo Fisher). Lysates (500 μL) were precleaned with Pierce NeutrAvidin Agarose (50 μL, Thermo Fisher) overnight with constant rotation at 4 °C to reduce endogenous biotinylation. Protein concentrations were quantified via Pierce bicinchoninic acid (BCA) assay (Thermo Fisher) and normalized using RIPA buffer. CuAAC was performed on 187.5 μL of lysate with final concentrations of 1 mM copper(II) sulfate (4 μL), 100 μM TBTA (3.12 μL), 1 mM sodium ascorbate (3.4 μL), and 100 μM AzTB (2 μL) at 37 °C for 2 h with constant shaking with final volume of 200 μL. Proteins were precipitated with chloroform/methanol (1:1 v/v) at 4 °C to remove excess fluorophore and lipids. Protein pellets were then resuspended in 200 μL of RIPA buffer. Fifteen μL of each sample was saved as pre-enrichment click input and the remaining 185 μL of clicked lysate was incubated with 40 μL of NeutrAvidin Agarose beads. Beads were washed (5× with RIPA buffer and 5× with PBS), and biotinylated proteins were eluted in 30 μL of 4× Laemmli buffer (BioRad) and boiled (95 °C for 15 min). Inputs (∼10–15 μg) and eluted proteins (15 μL) were analyzed by SDS-PAGE for in-gel fluorescence and Coomassie staining using an Amersham ImageQuant 800 (Cytiva).

For immunoblotting, 15 μL of eluted proteins were resolved by 12% SDS-PAGE and transferred onto an Immuno-Blot PVDF membrane (Thermo Fisher) using the Trans-Blot Turbo Transfer System (Bio-Rad) at 25 V for 30 min. The PVDF membrane was blocked for 1 h at room temperature in 5% dry nonfat milk prepared in Tris-buffered saline containing 0.01% Tween 20 (TBST; 50 mM Tris, 0.5 M NaCl, pH 7.4), then incubated overnight at 4 °C with constant shaking with primary antibodies. Primary antibodies that were diluted in blocking buffer included mouse monoclonal anti-Mincle (1:1000: MBL International) and rabbit recombinant anti-TREM2 (1:1000; Proteintech). After washing thrice with TBST, PVDF membranes were incubated with Horseradish peroxidase (HRP)-conjugated secondary antibodies diluted in TBST containing 5% milk (antirat IgG, Sigma; antirabbit IgG, Cell Signaling, 1:1000) for 1 h at room temperature. The PVDF membrane was then washed thrice with TBST and viewed with the Amersham ImageQuant 800.

## Supporting Information Available

^1^H, ^13^C, and mass spectra of synthetic compounds (PDF)
