# Supplementary material for: A Photoactivatable Free Mycolic Acid Probe to Investigate Mycobacteria–Host Interactions
Source: ACS Infect Dis. 2025 Apr 14;11(5):1233–45. doi: 10.1021/acsinfecdis.5c00068 (PMC12070411; doi:10.1021/acsinfecdis.5c00068)

Supporting Information (SI) for:

A Photoactivatable Free Mycolic Acid Probe to Investigate Mycobacteria–Host Interactions

Kingsley C. Agu,[1,‡] Nicholas Banahene,[1,2,‡] Carolina Santamaria,[3] Christi Y. Kim,[4] Jessica Cabral,[4] Kyle J. Biegas,[1,2] Casey Papson,[1] Andrew D. Kruskamp,[1] M. Sloan Siegrist,[3,4] and Benjamin M. Swarts[1,2,*]

[1]Department of Chemistry and Biochemistry, Central Michigan University, Mount Pleasant, MI, USA

[2]Biochemistry, Cell, and Molecular Biology Graduate Programs, Central Michigan University, Mount Pleasant, MI, 48859 USA

[3]Molecular and Cellular Biology Program, University of Massachusetts, Amherst, MA, 01003 USA

[4]Department of Microbiology, University of Massachusetts, Amherst, MA, 01003 USA

[‡]These authors contributed equally to this work.

*Corresponding author: E-mail: ben.swarts@cmich.edu

SI Table of Contents

I. NMR and MS spectra ____________ S2

I. NMR and MS Spectra

Compound **4** ^{1}H NMR

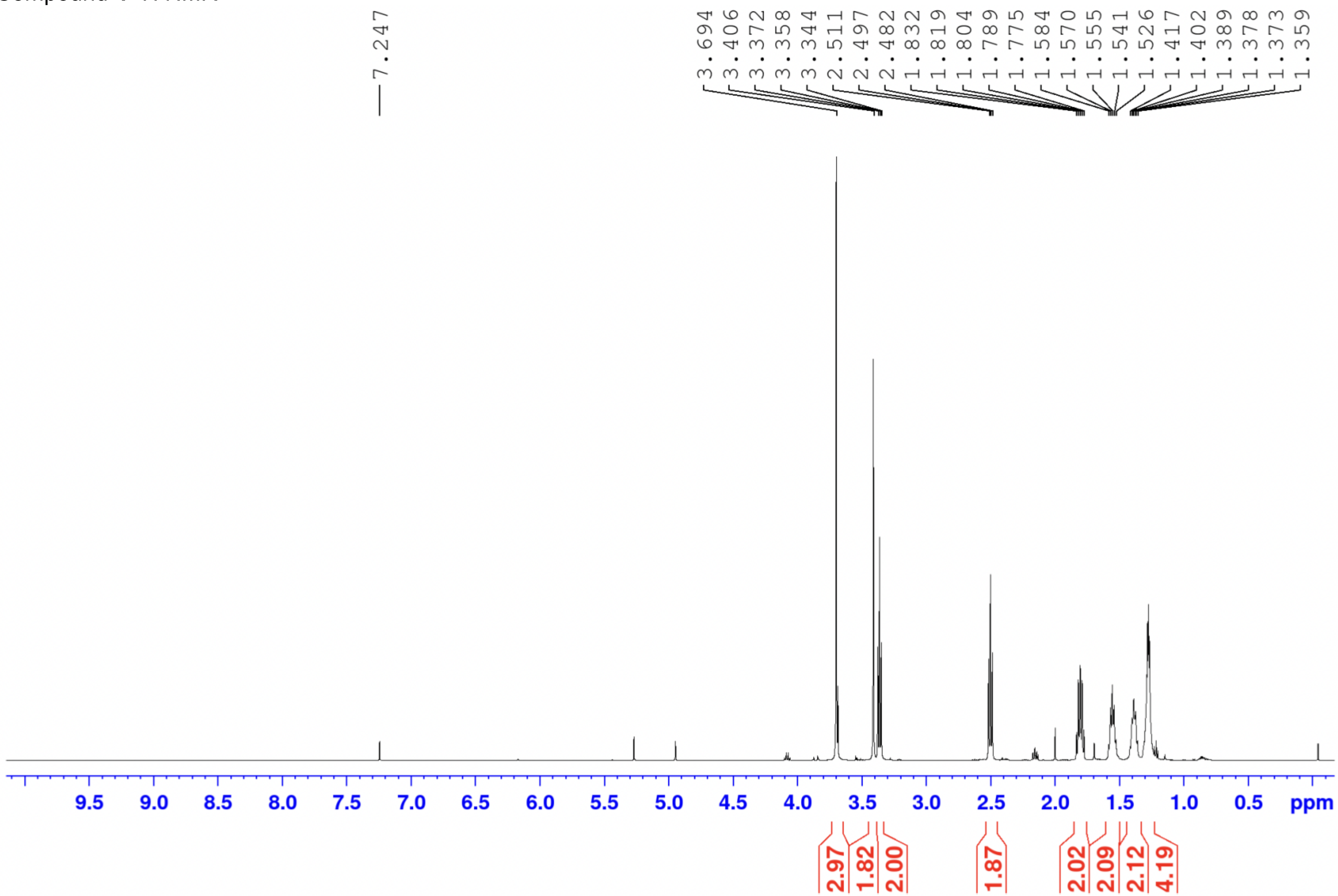

Compound **4** ^{13}C NMR

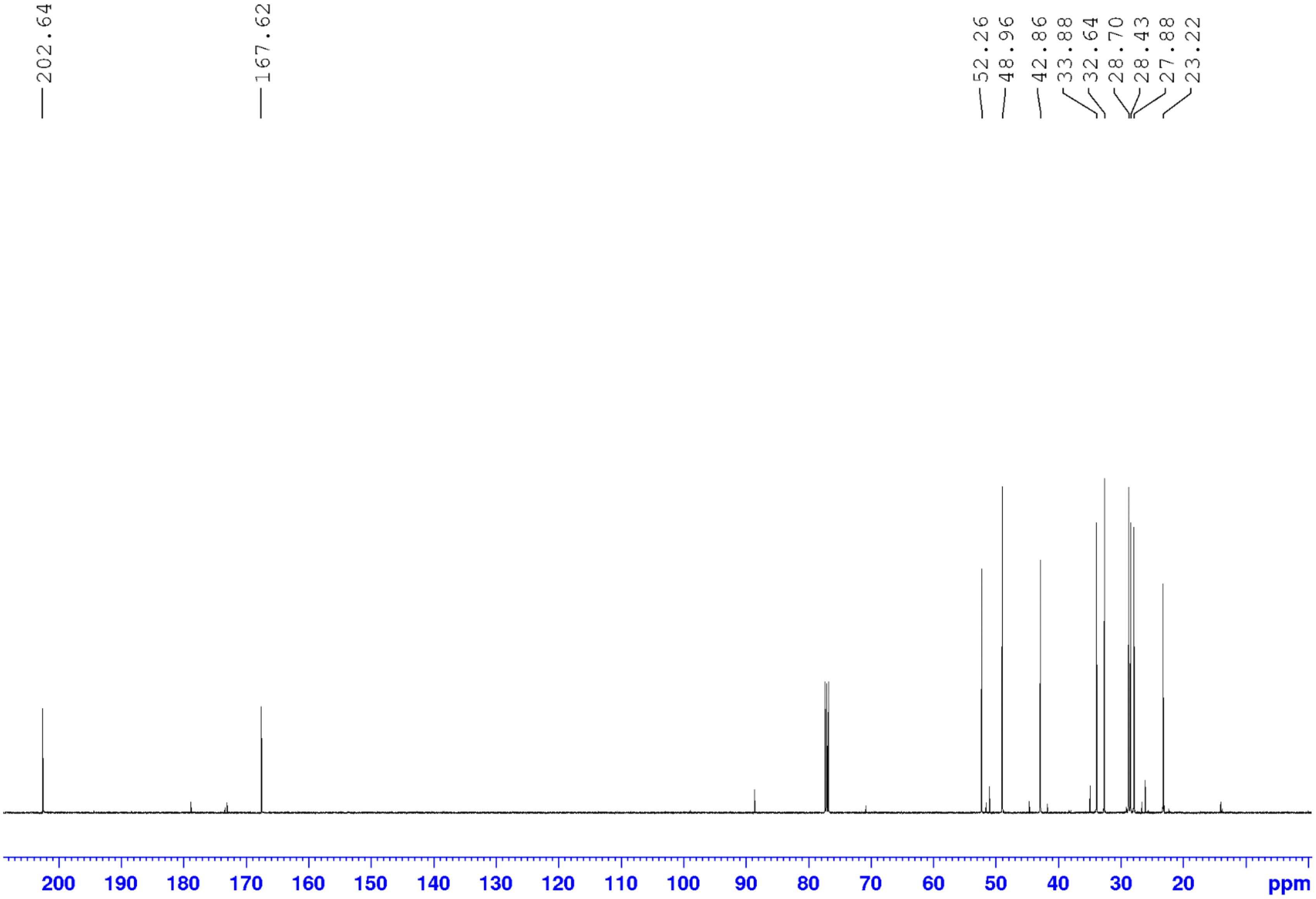

Compound **4** COSY NMR

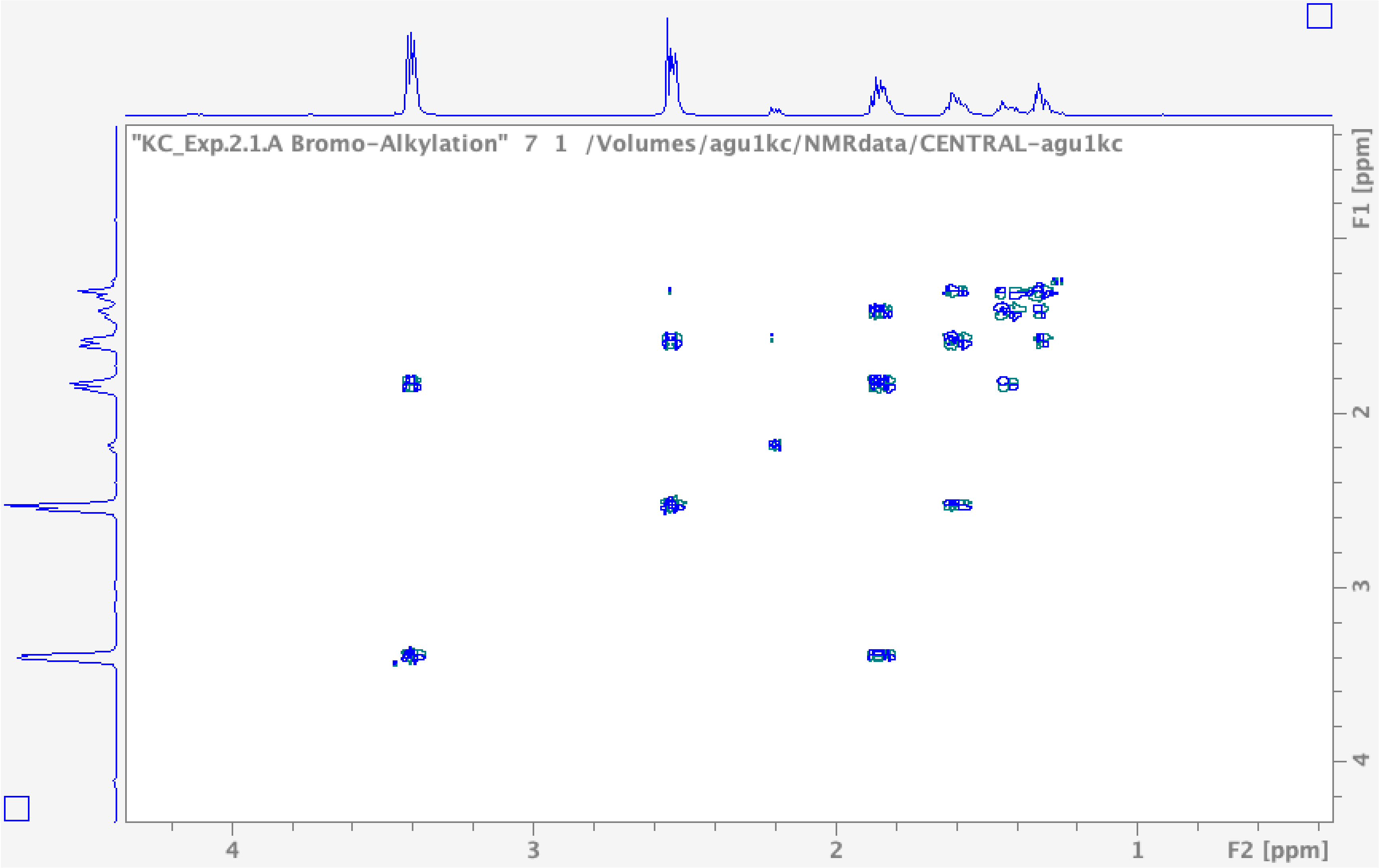

Compound **4** ESI MS

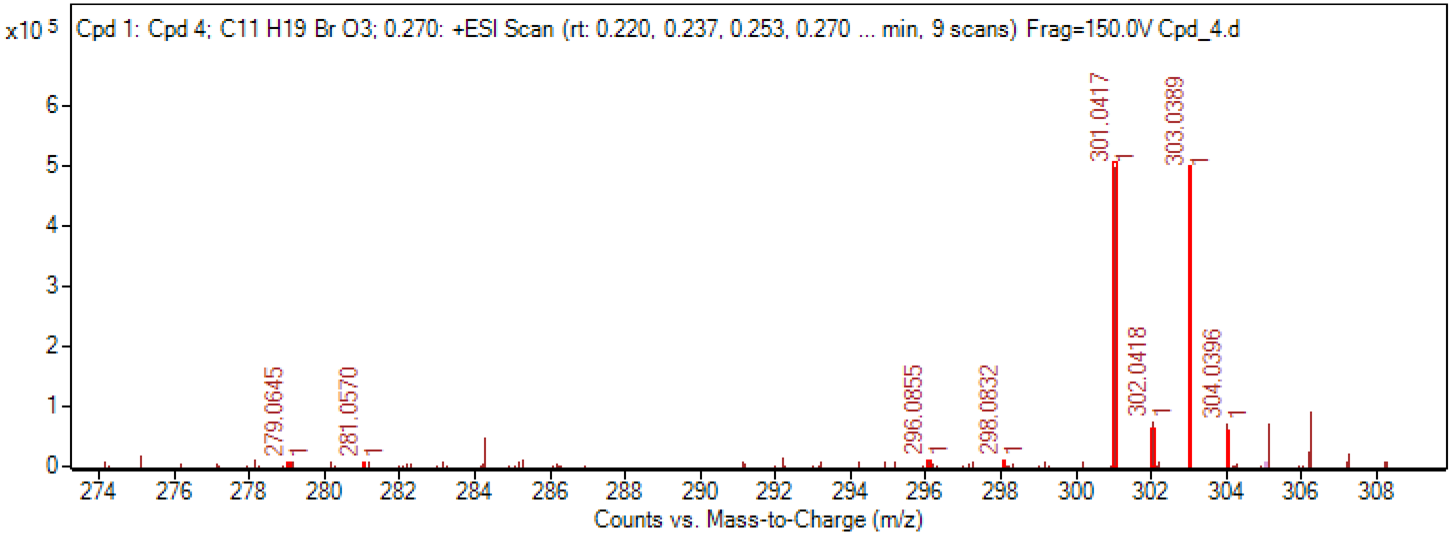

Compound **5** ^{1}H NMR

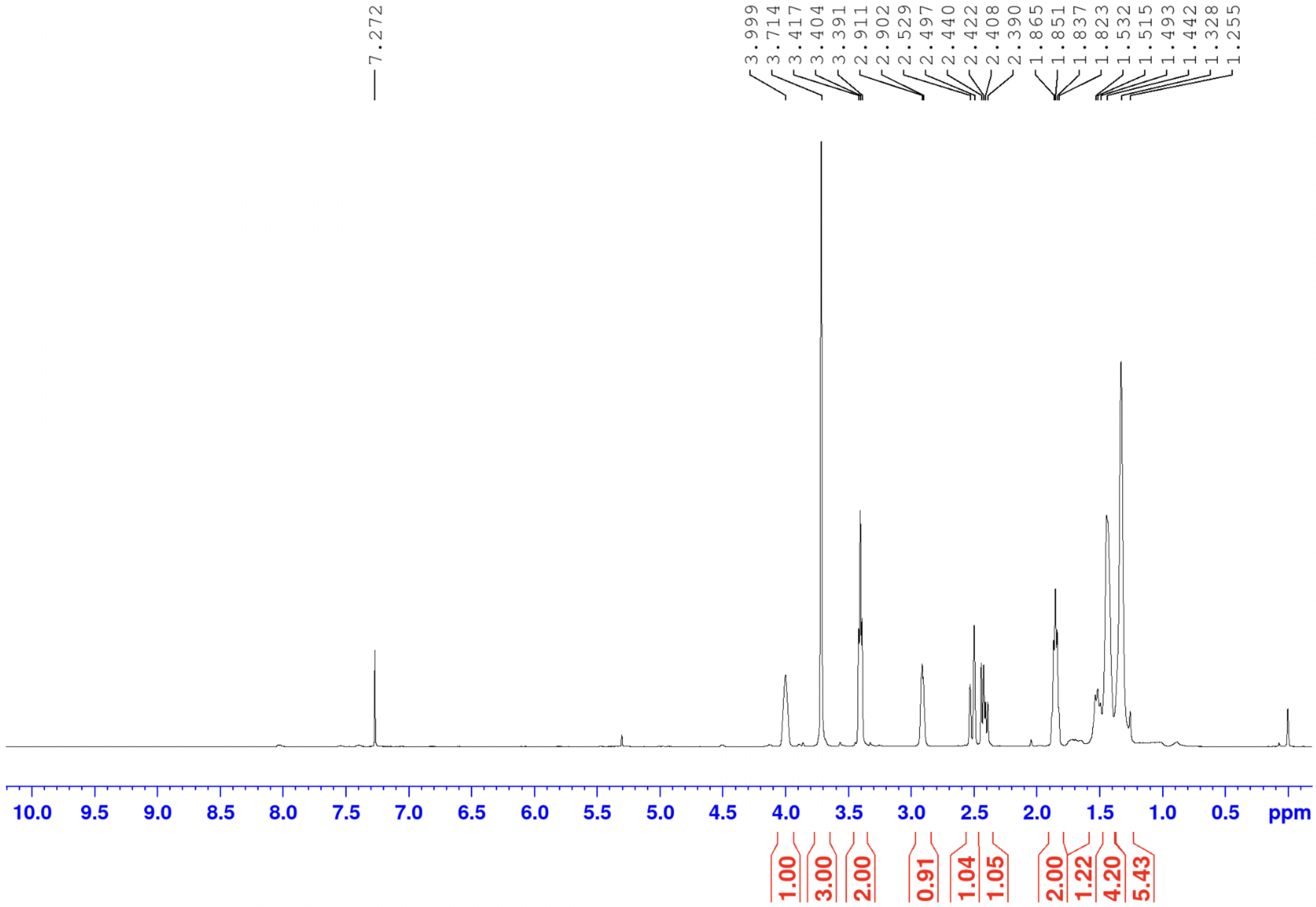

Compound **5** ^{13}C NMR

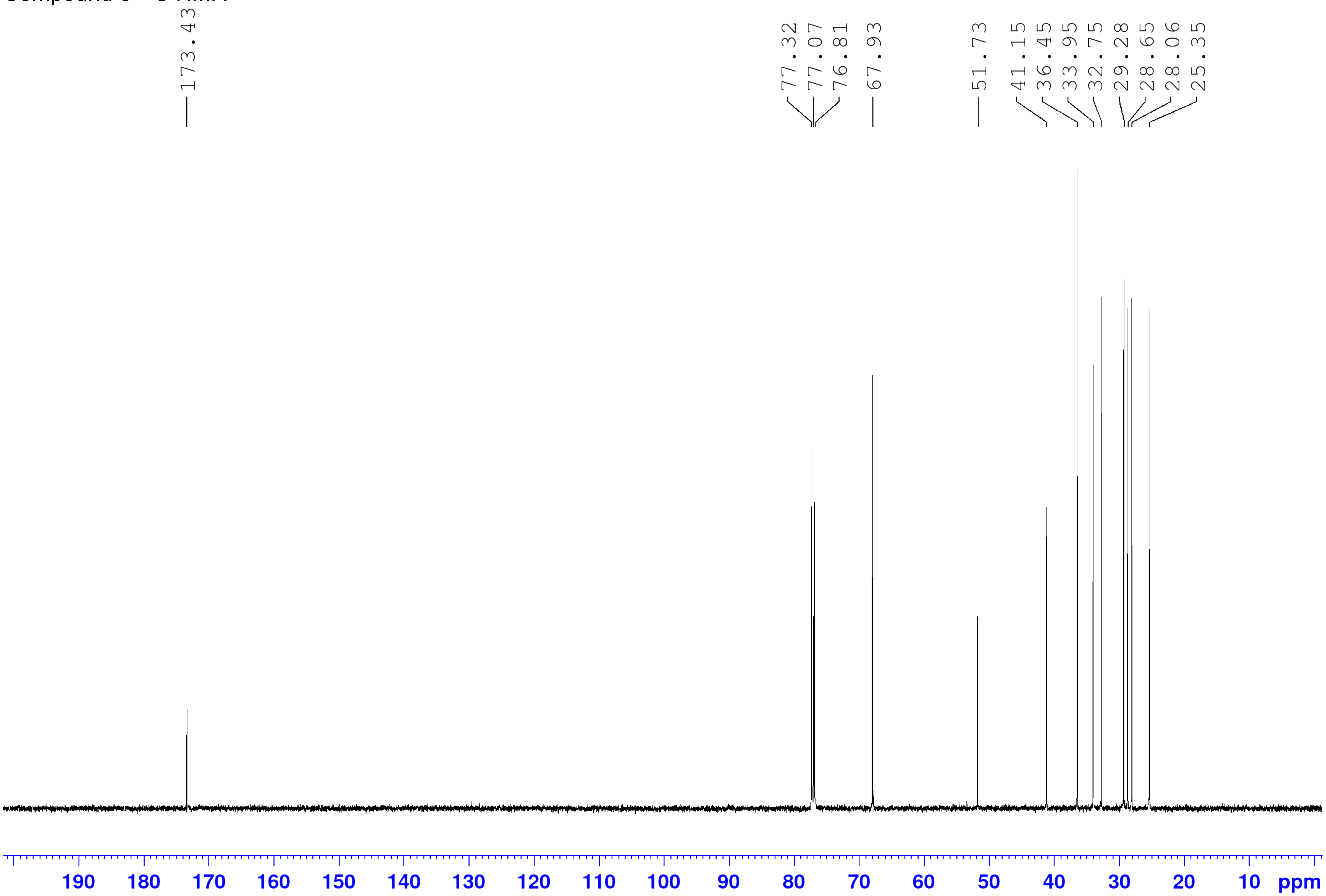

Compound **5** COSY NMR

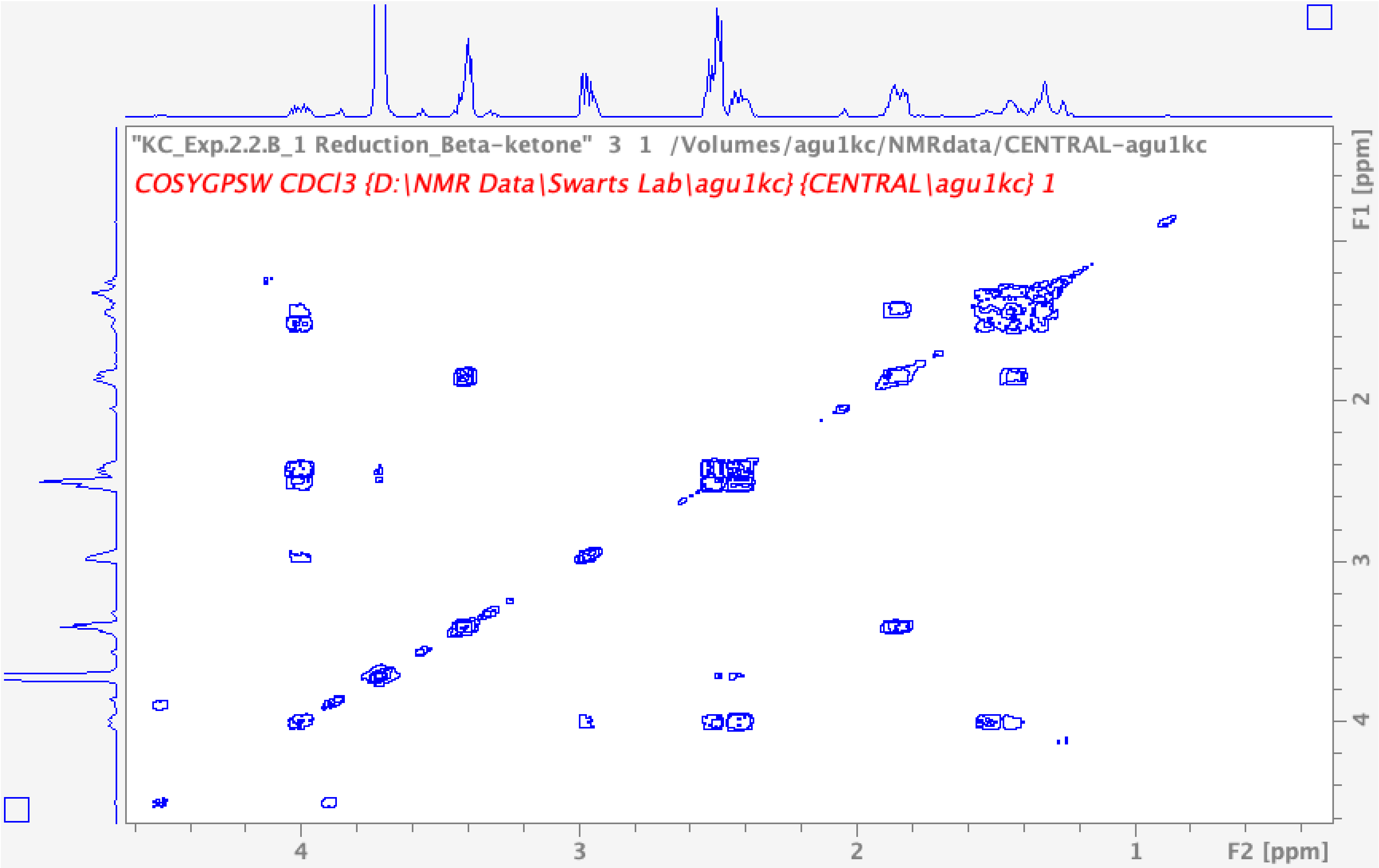

Compound **5** ESI MS

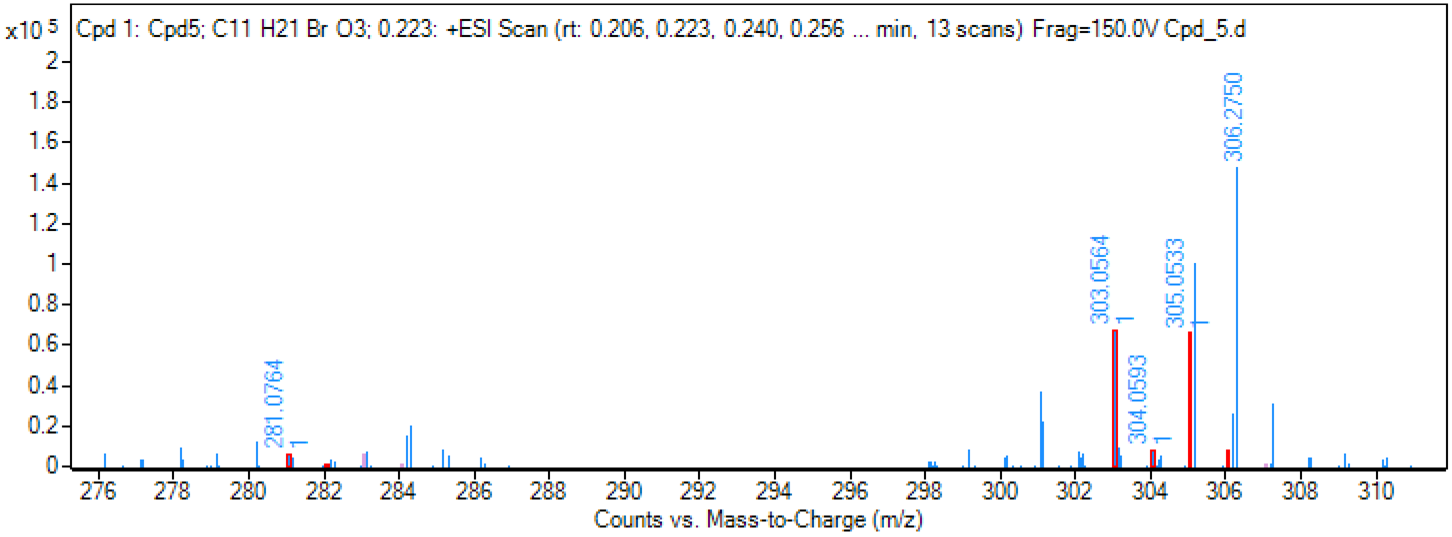

Compound (*R*)-MTPA-**5** ^{1}H NMR

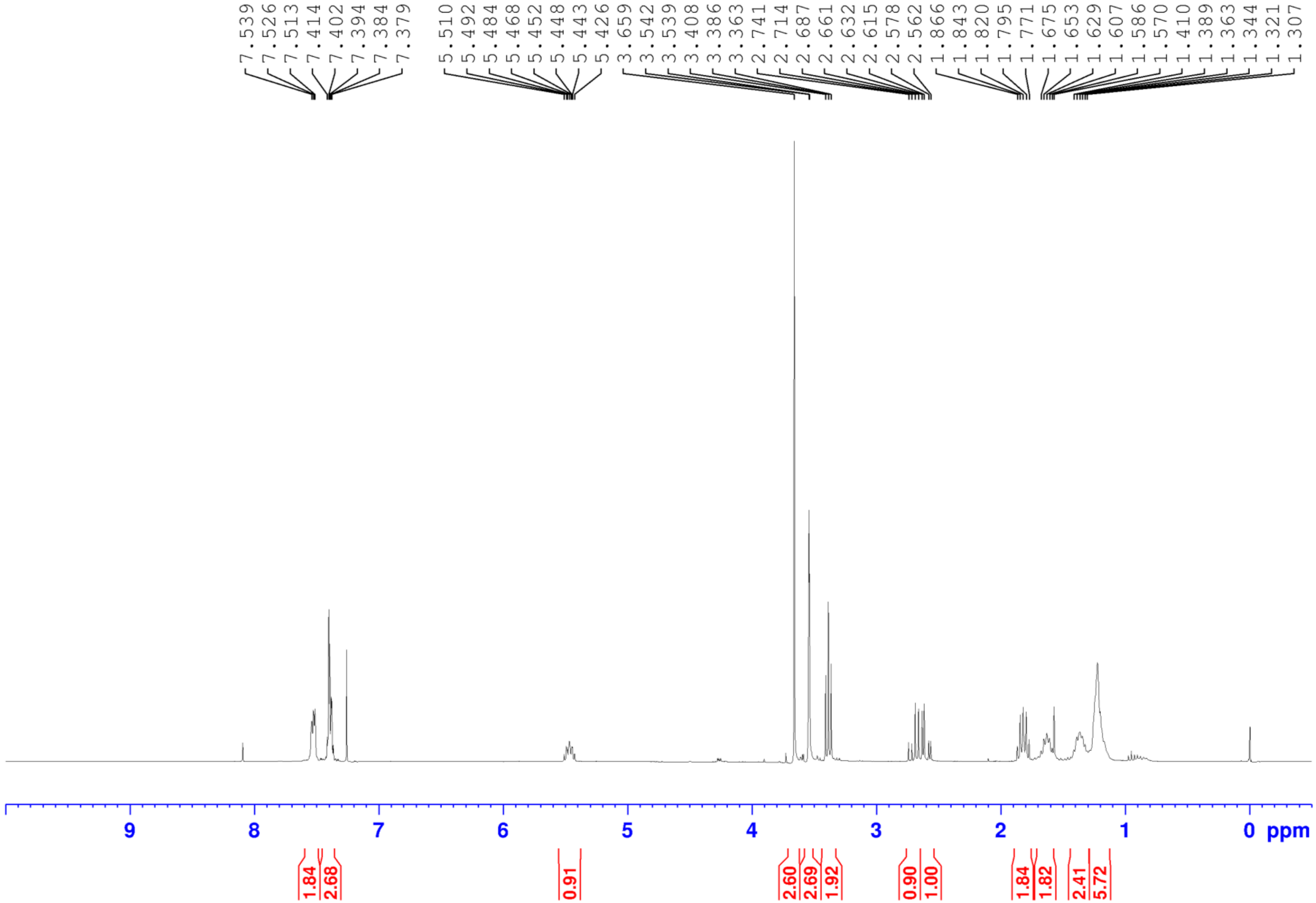

Compound (*S*)-MTPA-**5** ^{1}H NMR

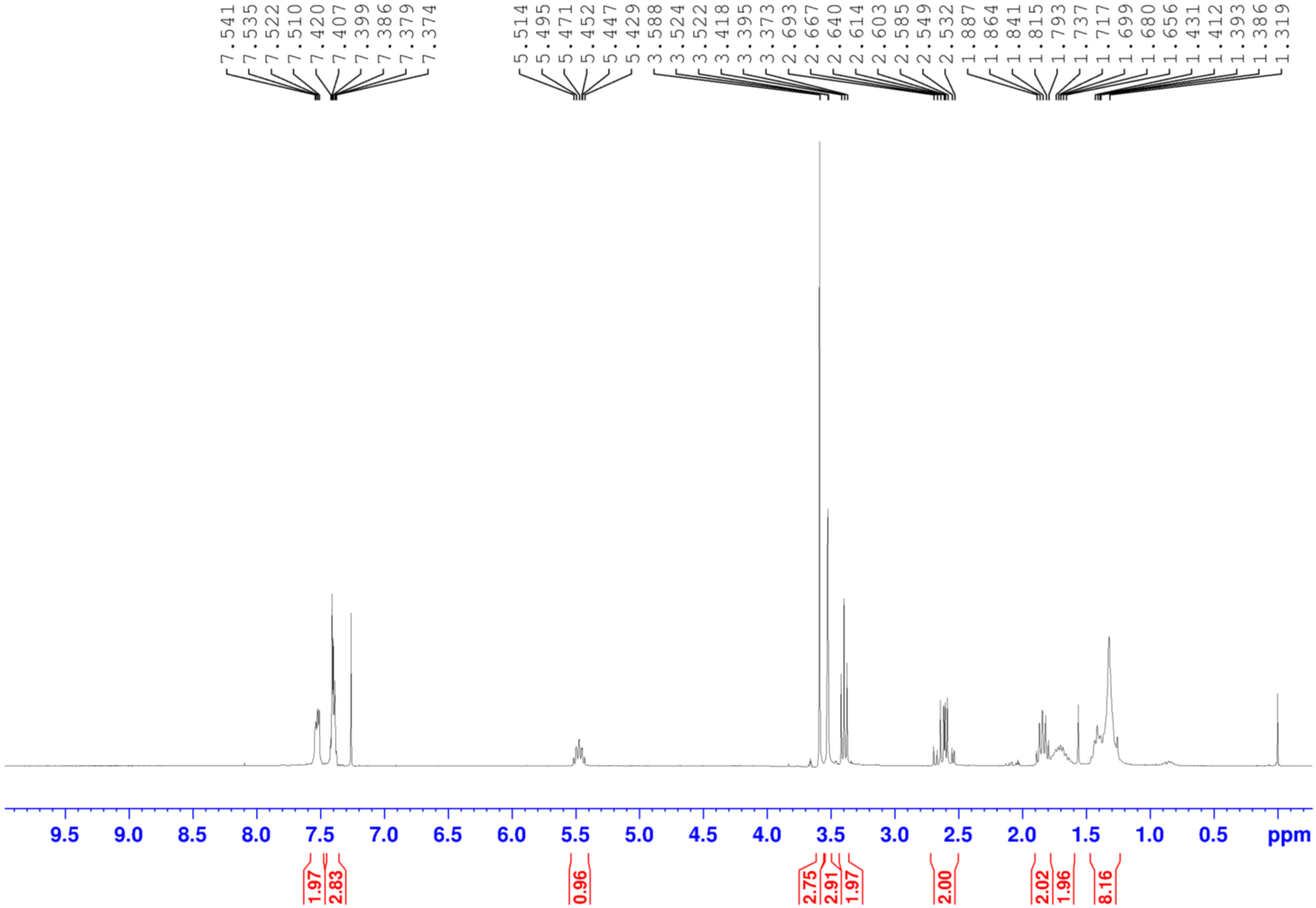

Compound (*R*)-MTPA-*Rac*-**5** ^{1}H NMR

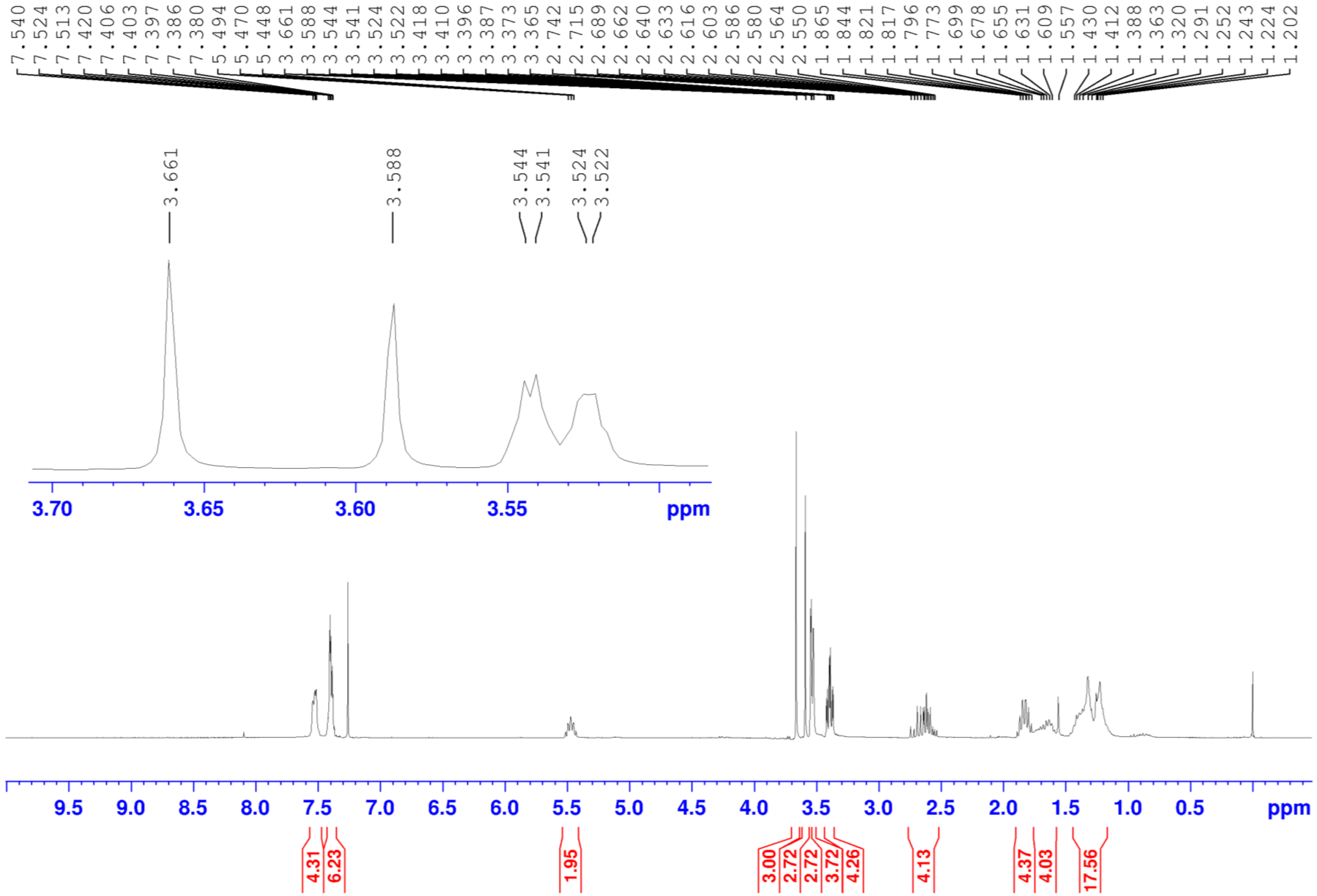

Compound **6** ^{1}H NMR

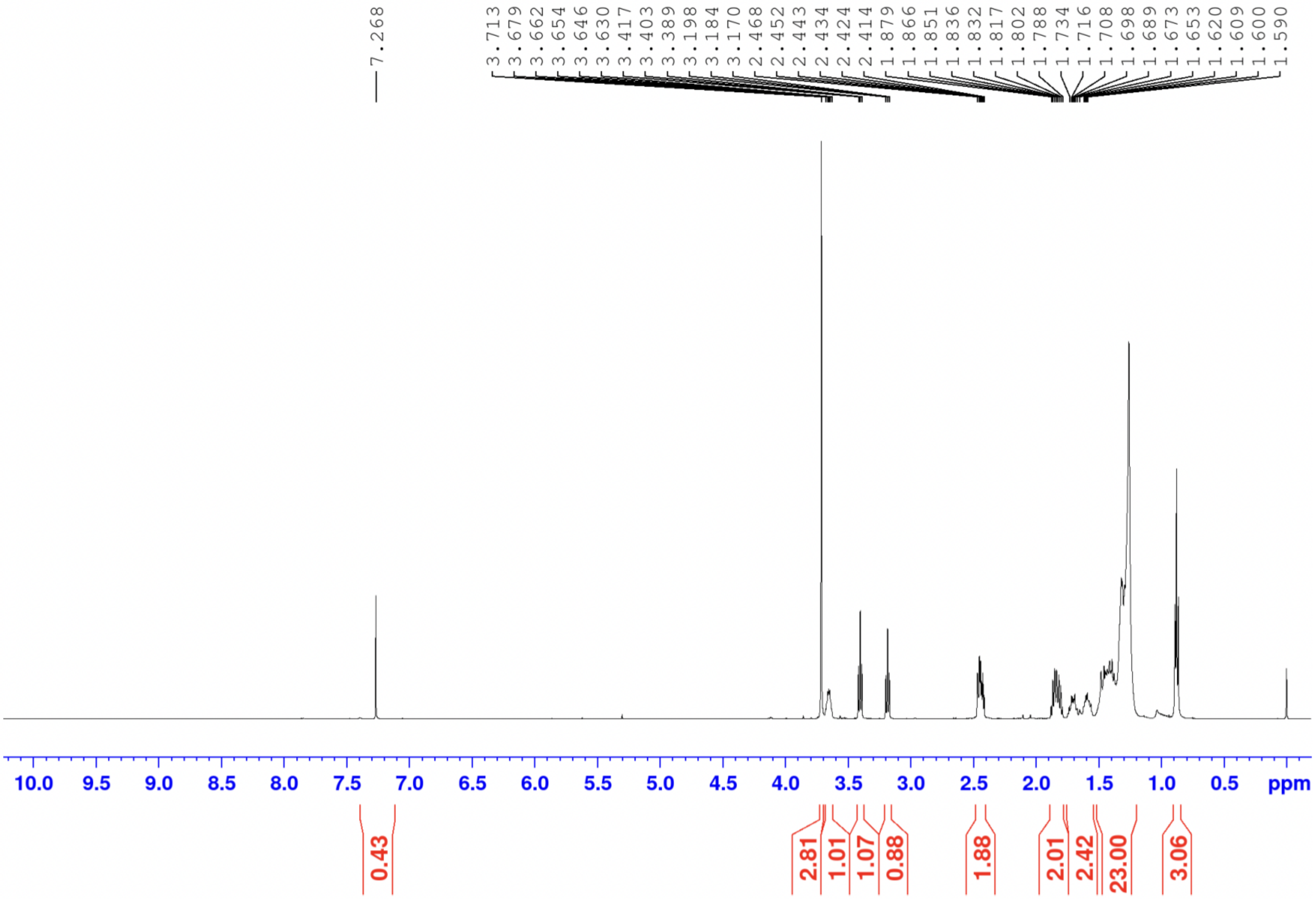

Compound **6** ^{13}C NMR

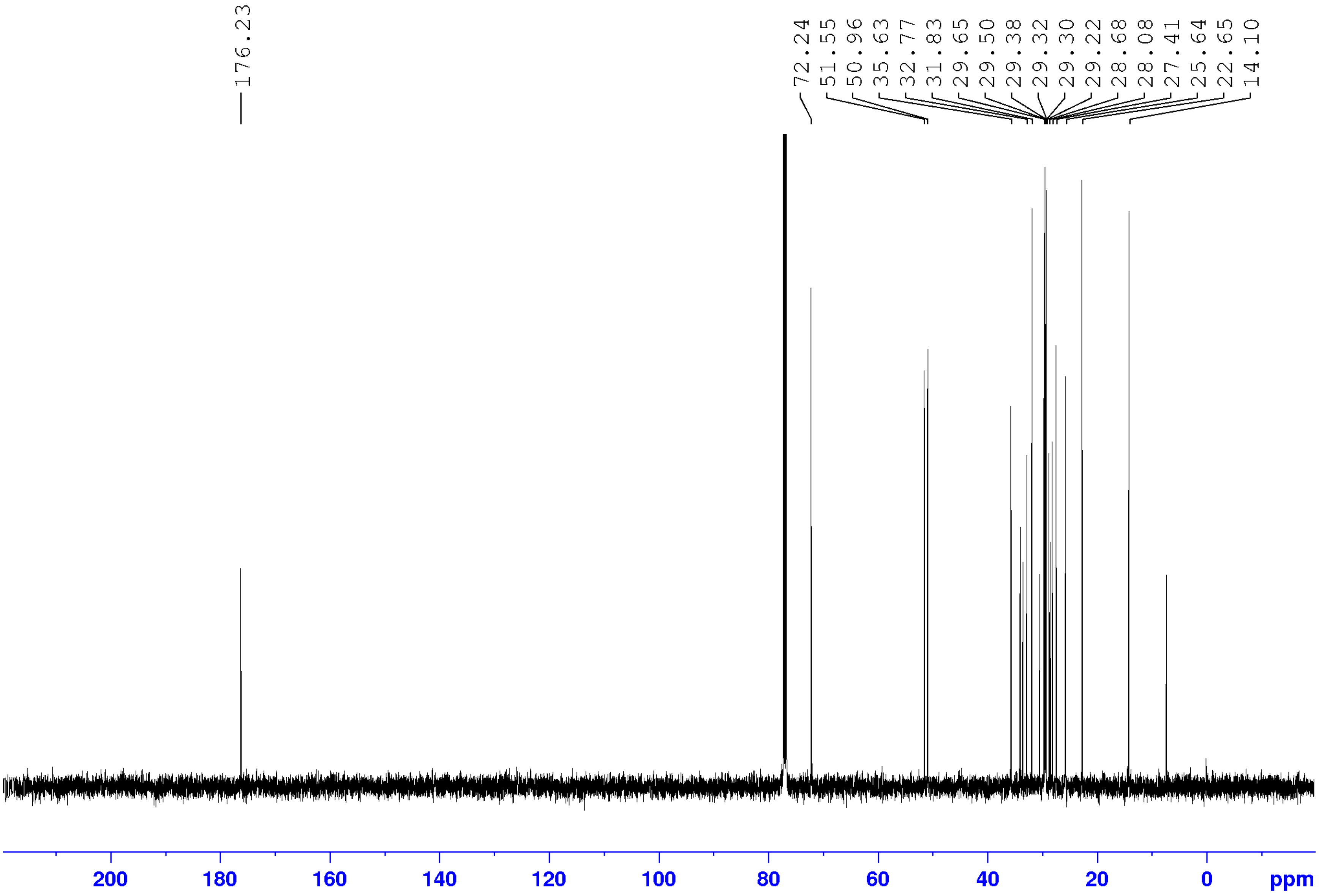

Compound **6** COSY NMR

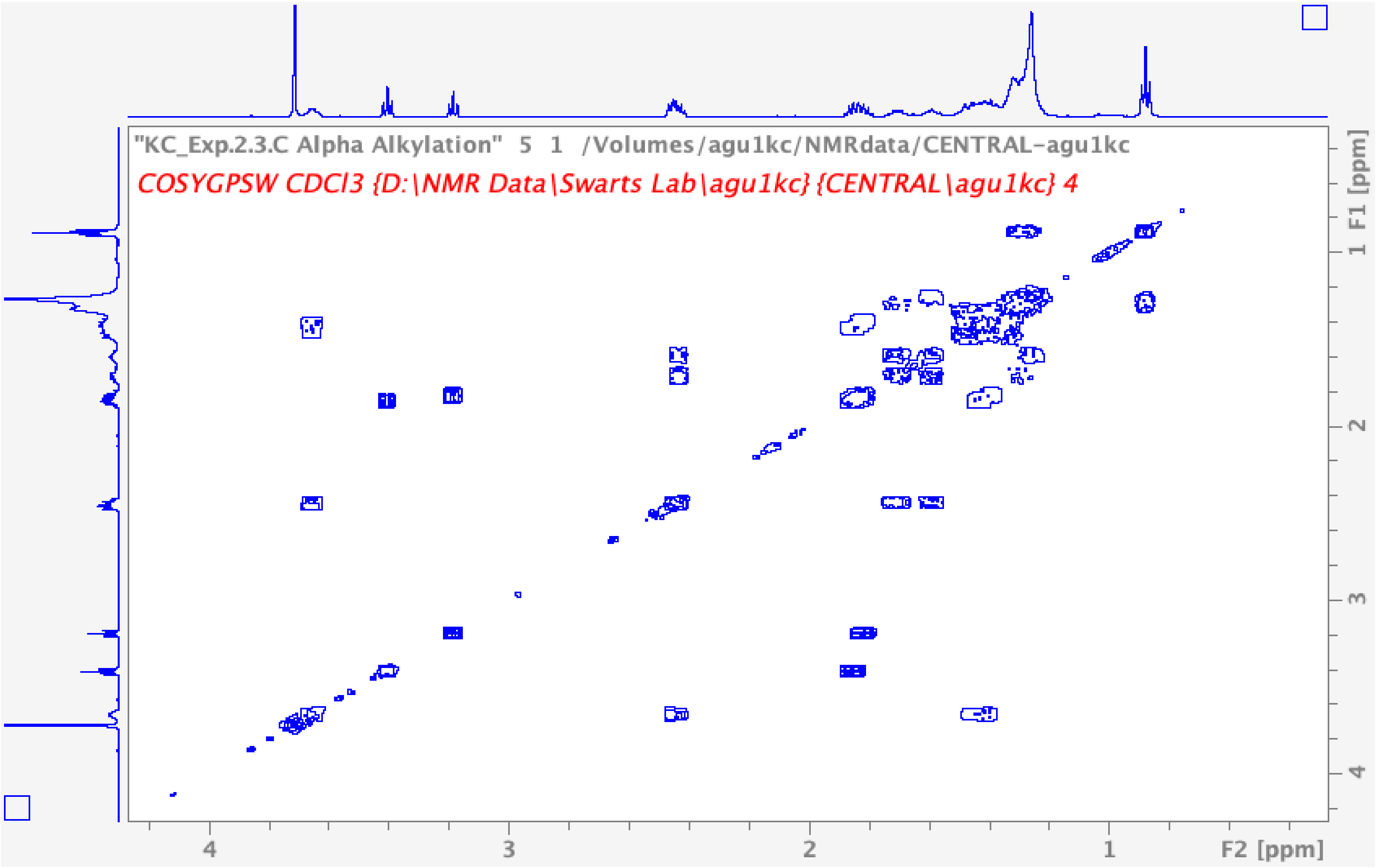

Compound **6** ESI MS

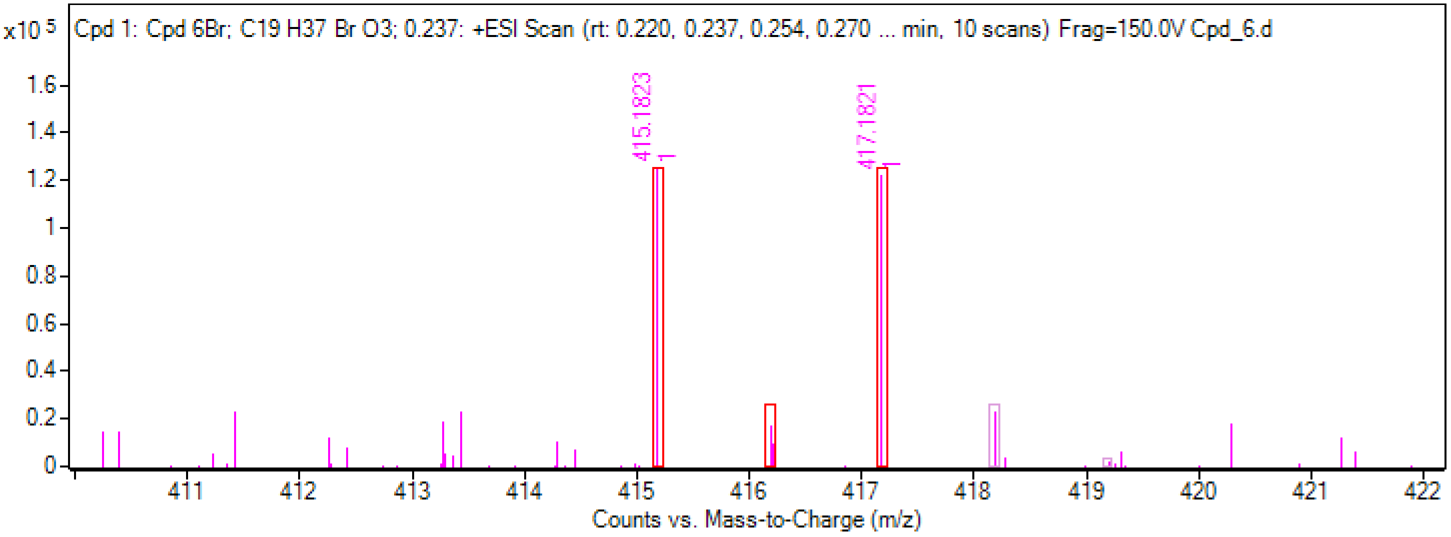

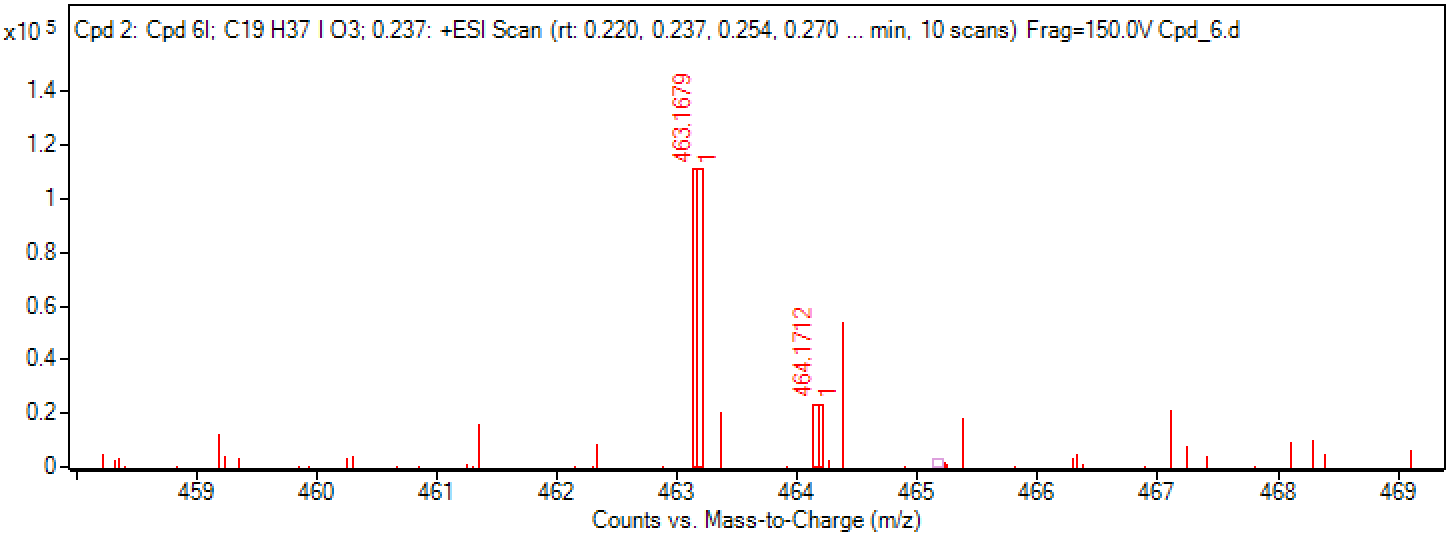

Compound **7** ^{1}H NMR

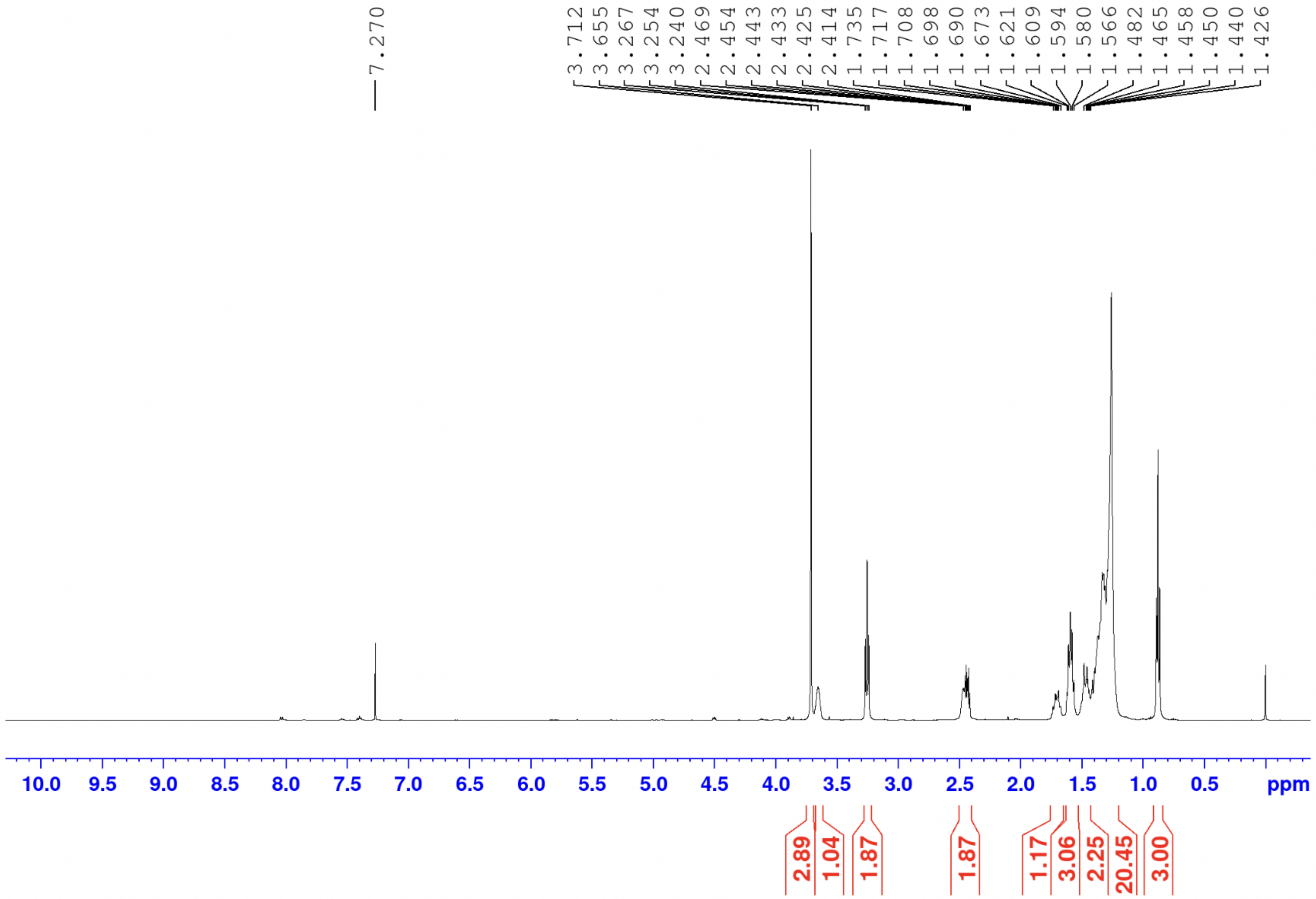

Compound **7** ^{13}C NMR

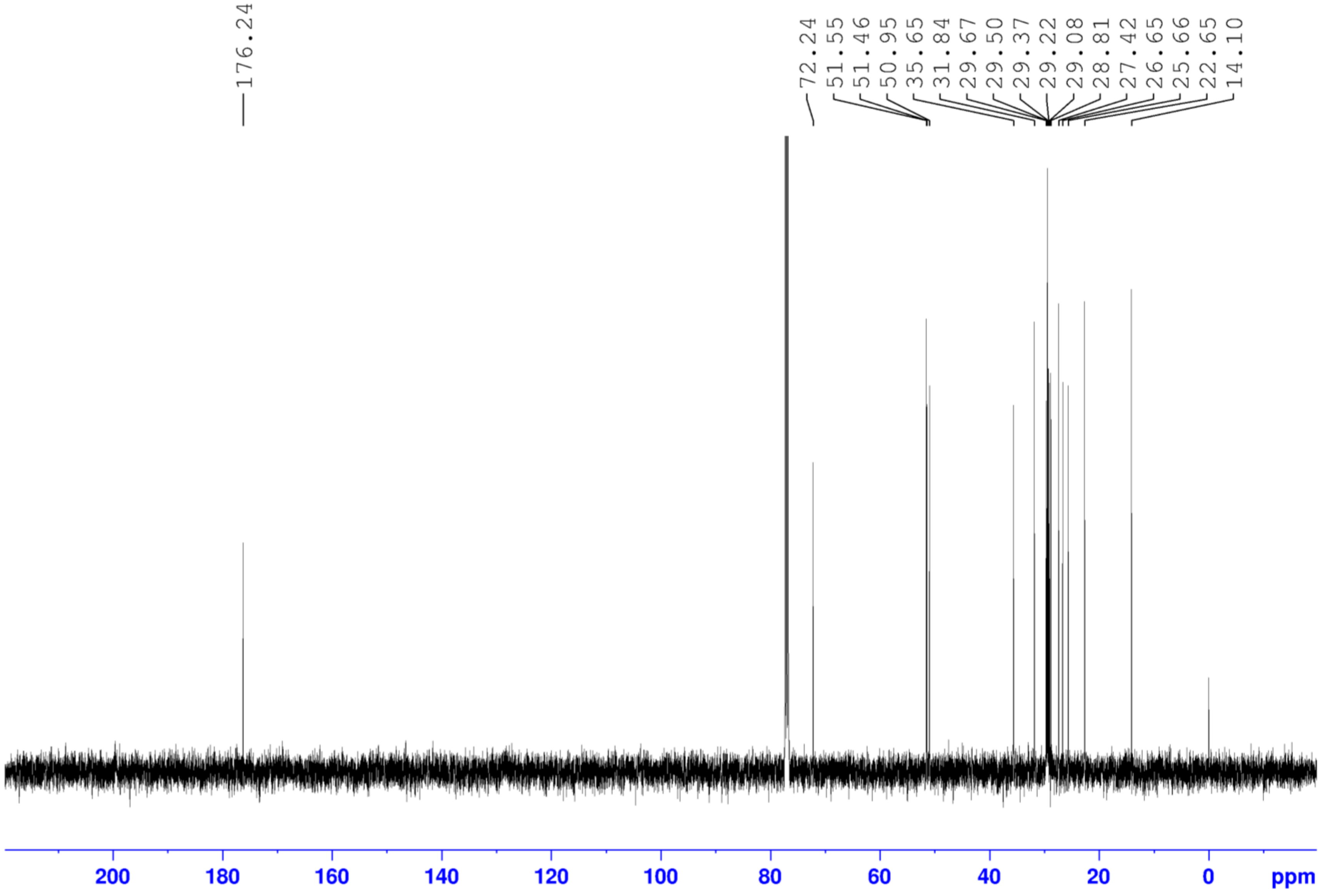

Compound **7** COSY NMR

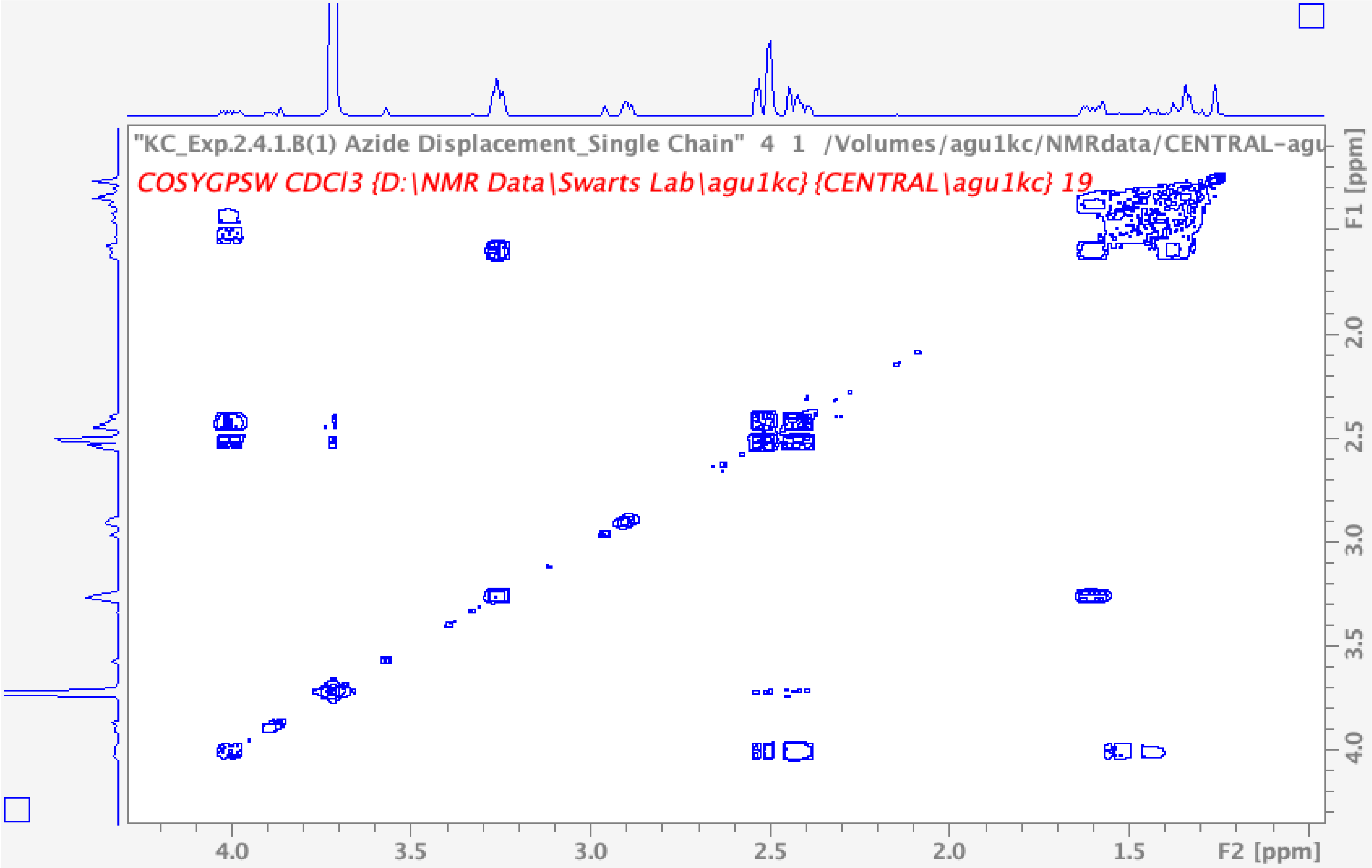

Compound **7-acetonide** ^{1}H NMR

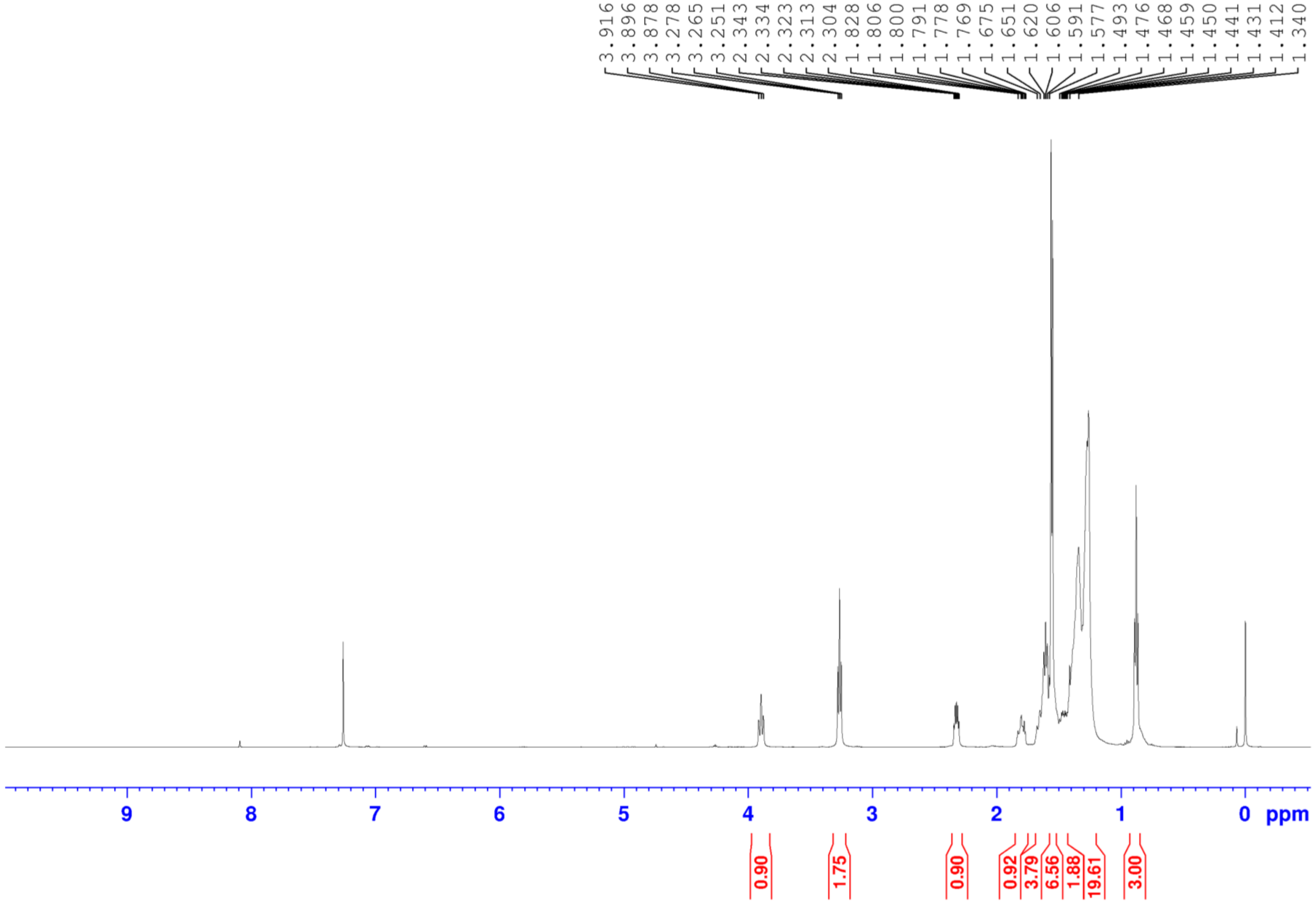

Compound **7-acetonide** ^{13}C NMR

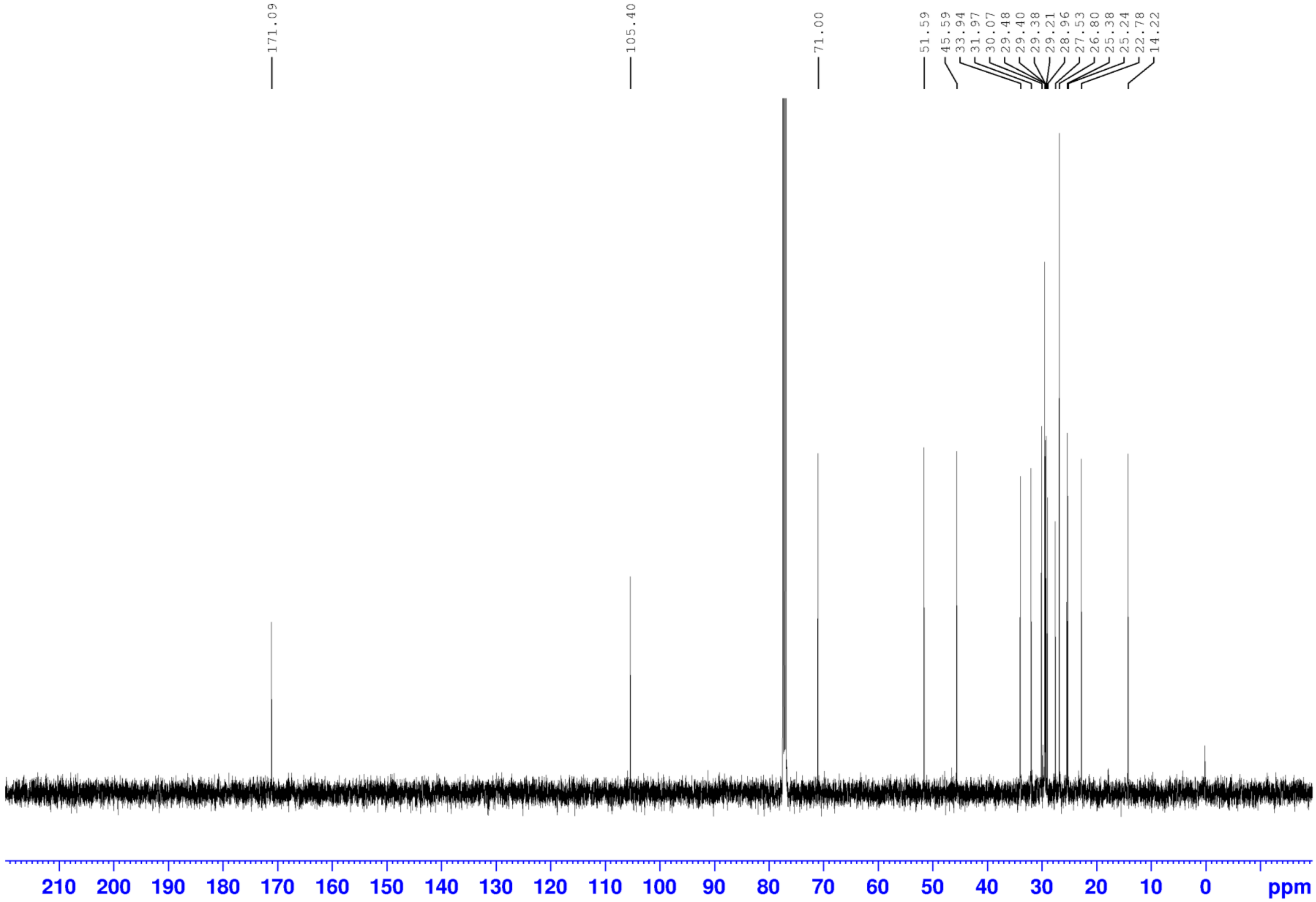

Compound **10** ^{1}H NMR

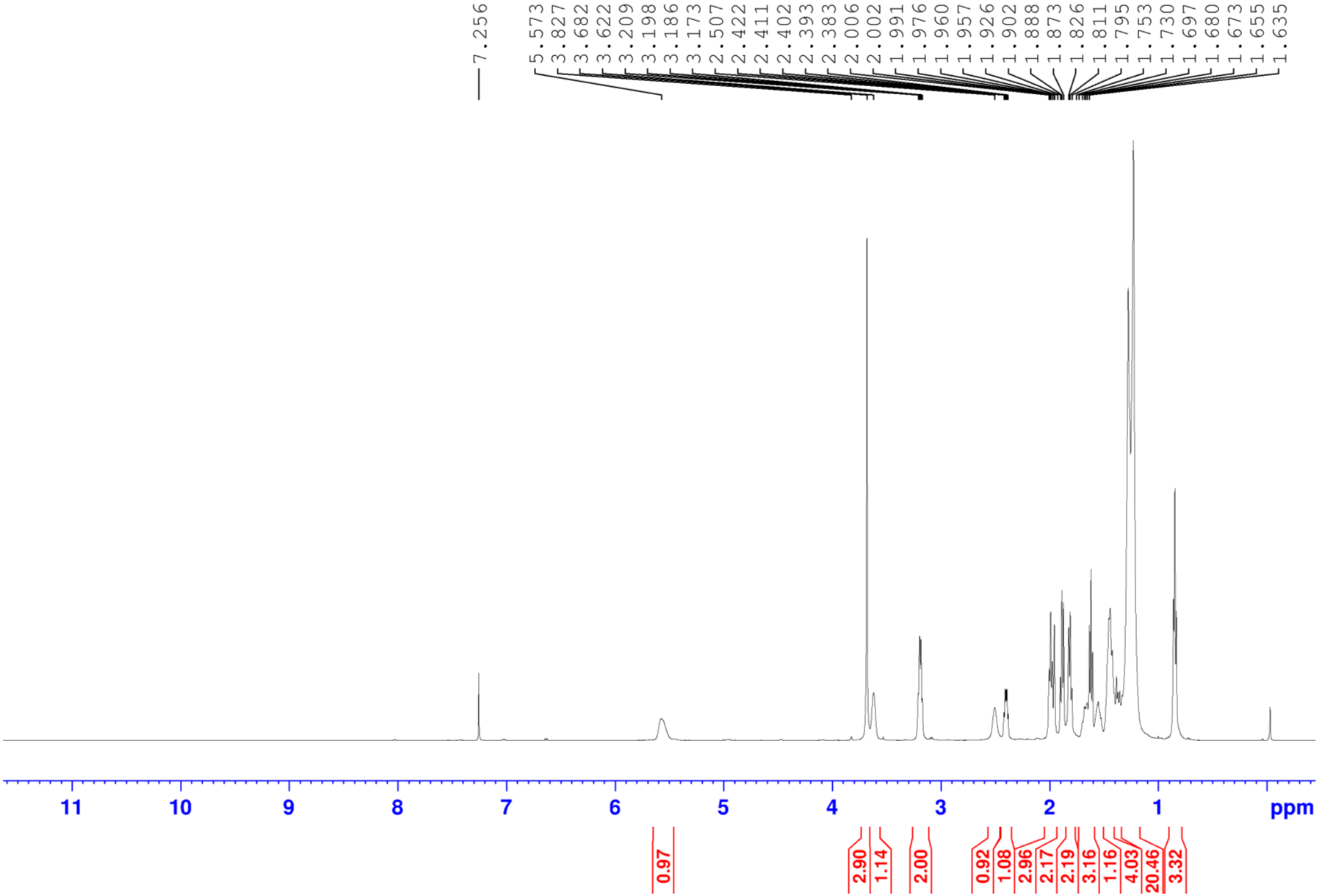

Compound **10** ^{13}C NMR

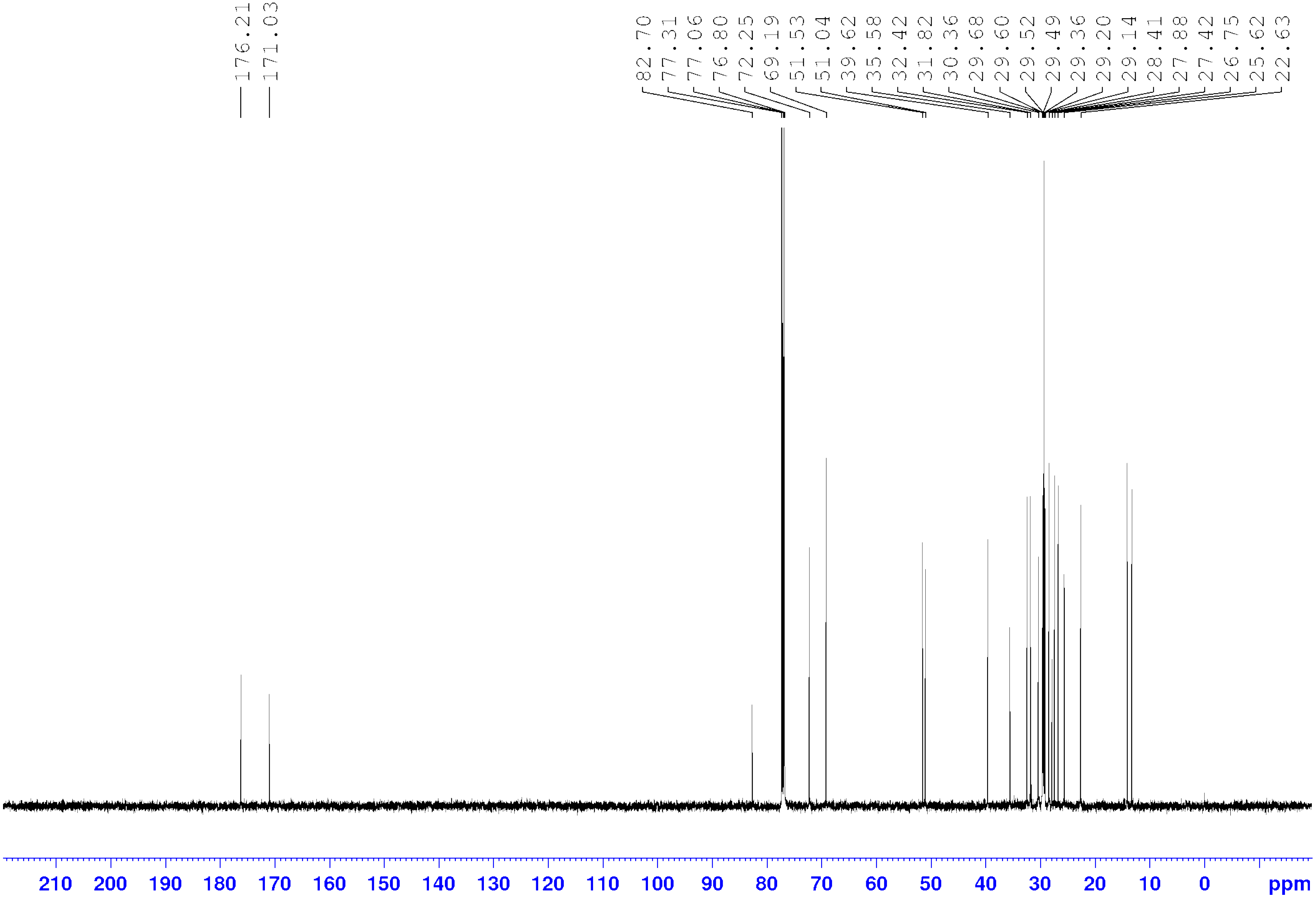

Compound **10** COSY NMR

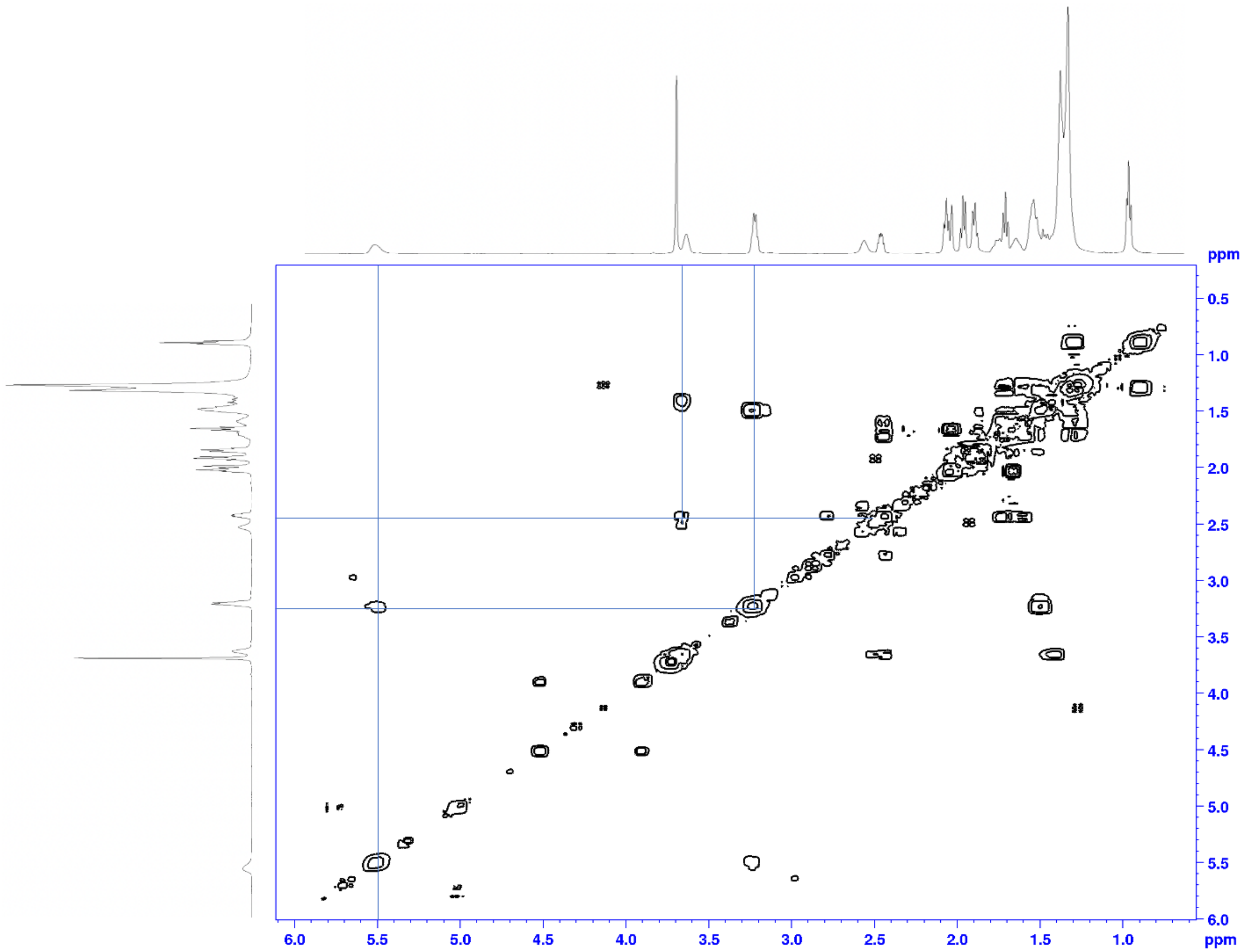

Compound **10** ESI MS

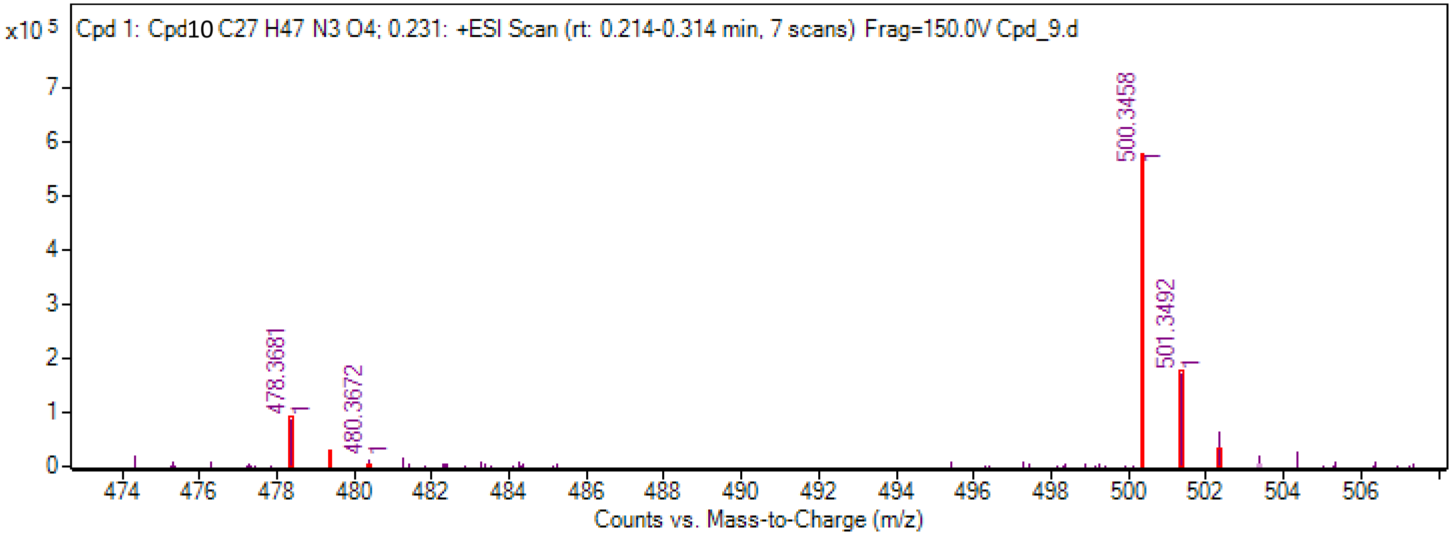

Compound **1** (**x-Alk-MA**) ^{1}H NMR

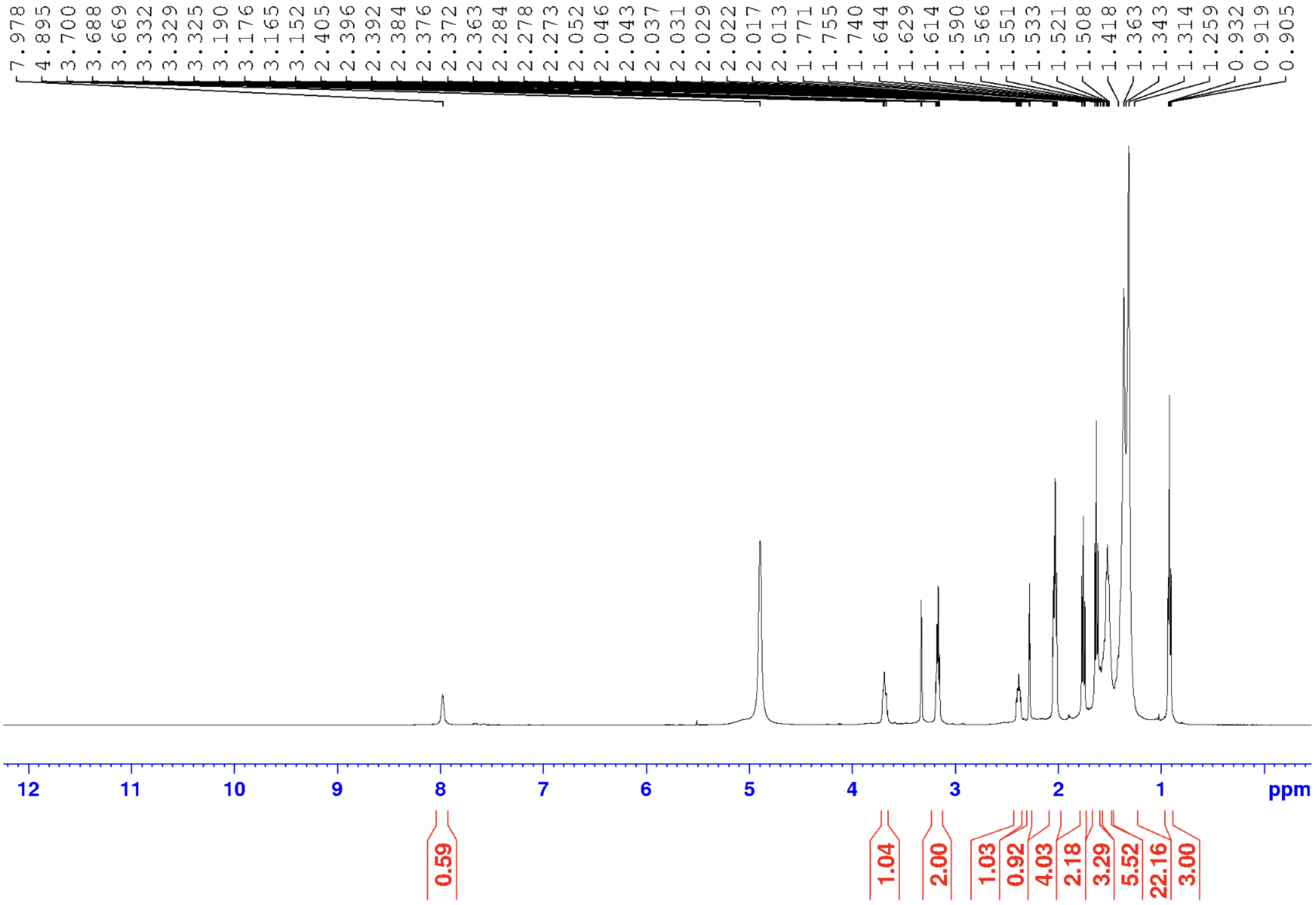

Compound **1** (**x-Alk-MA**) ^{13}C NMR

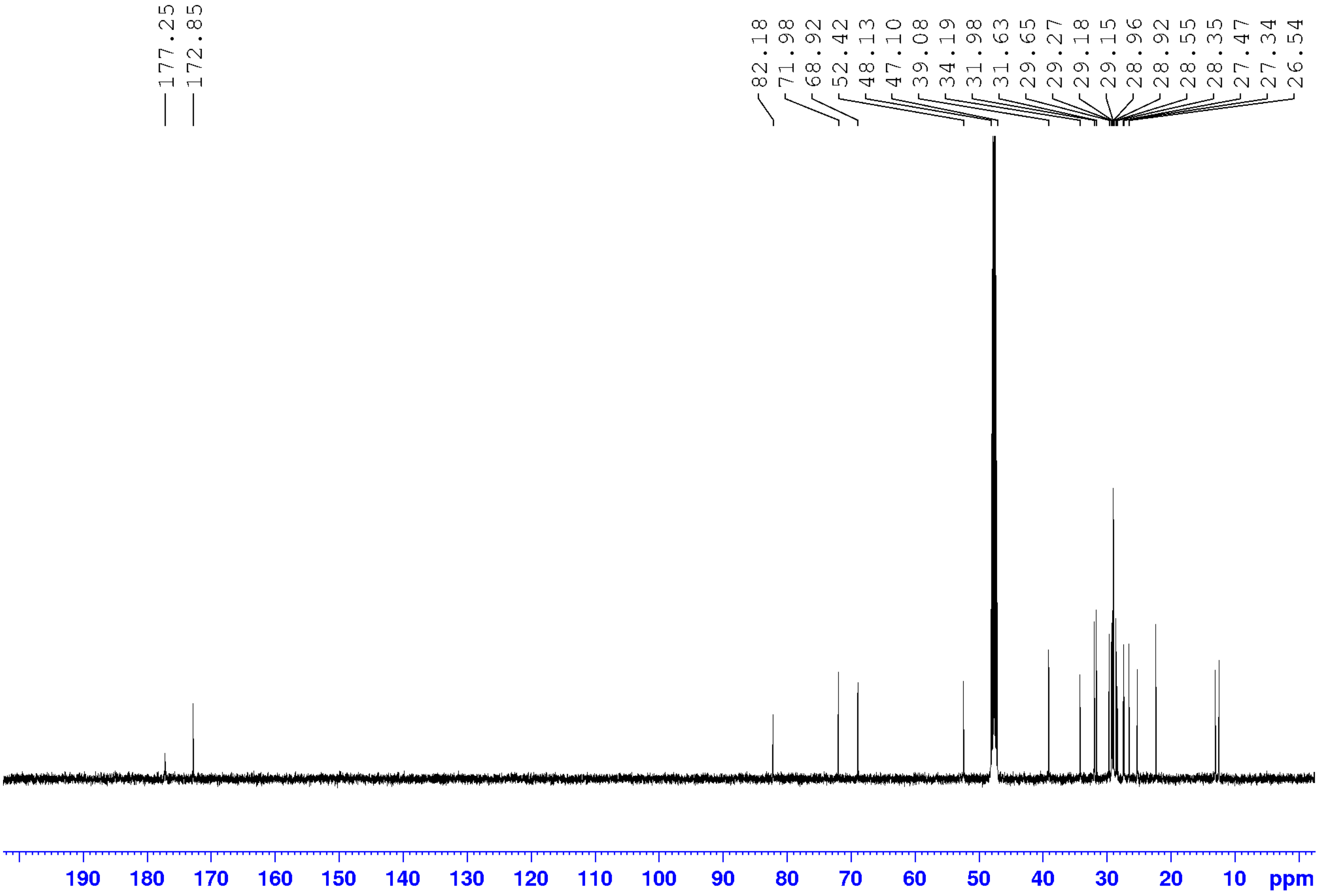

Compound **1** (**x-Alk-MA**) COSY NMR

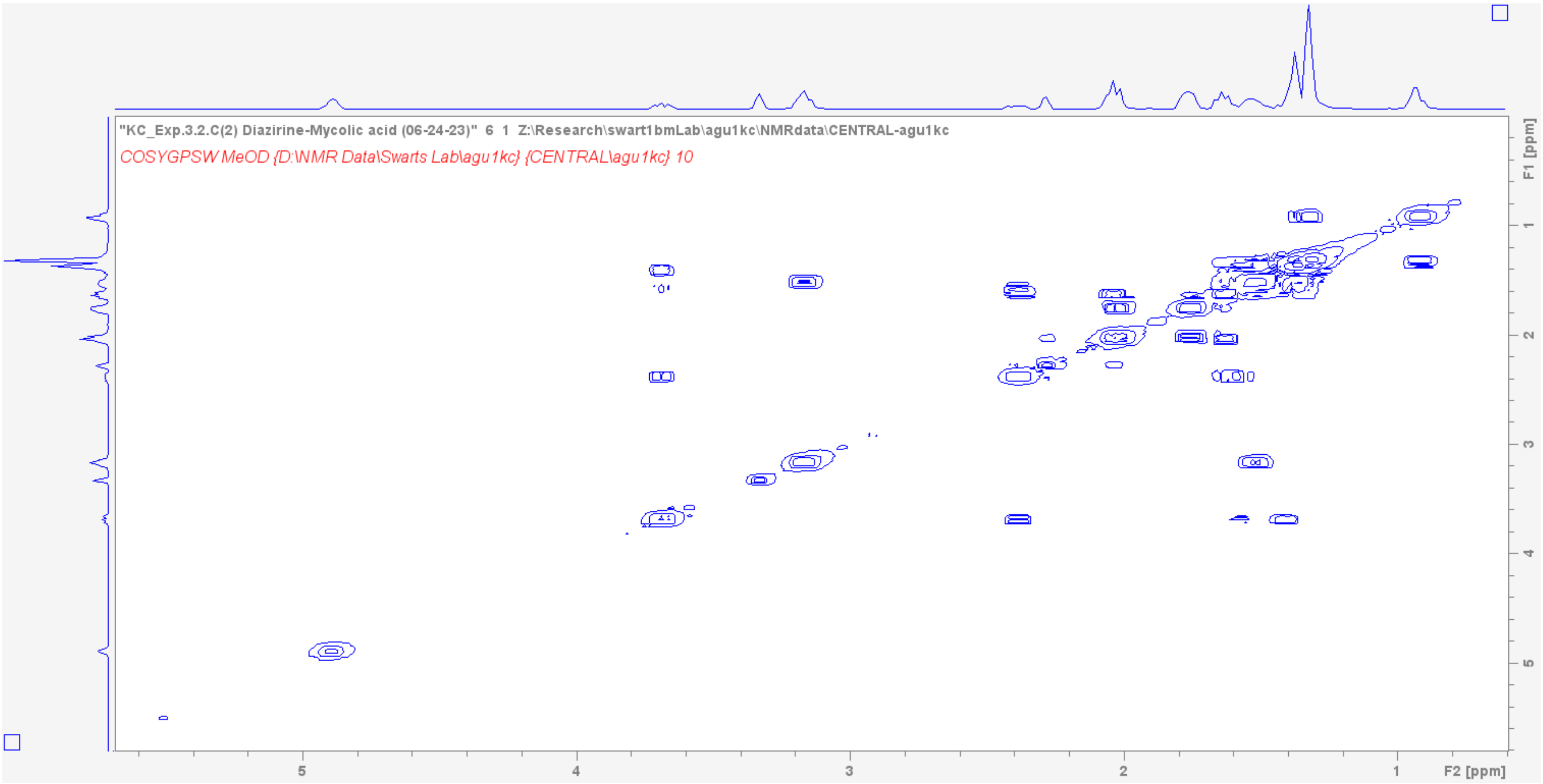

Compound **1** (**x-Alk-MA**) ESI MS

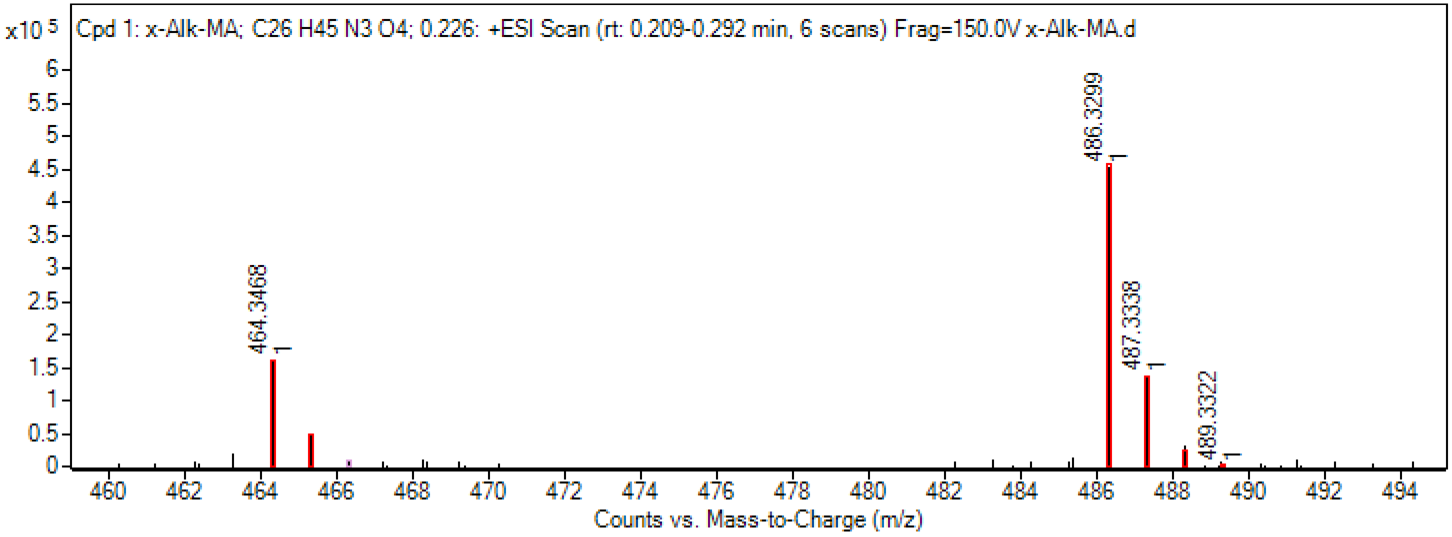

Compound **2** (**x-Alk-FA**) ^{1}H NMR

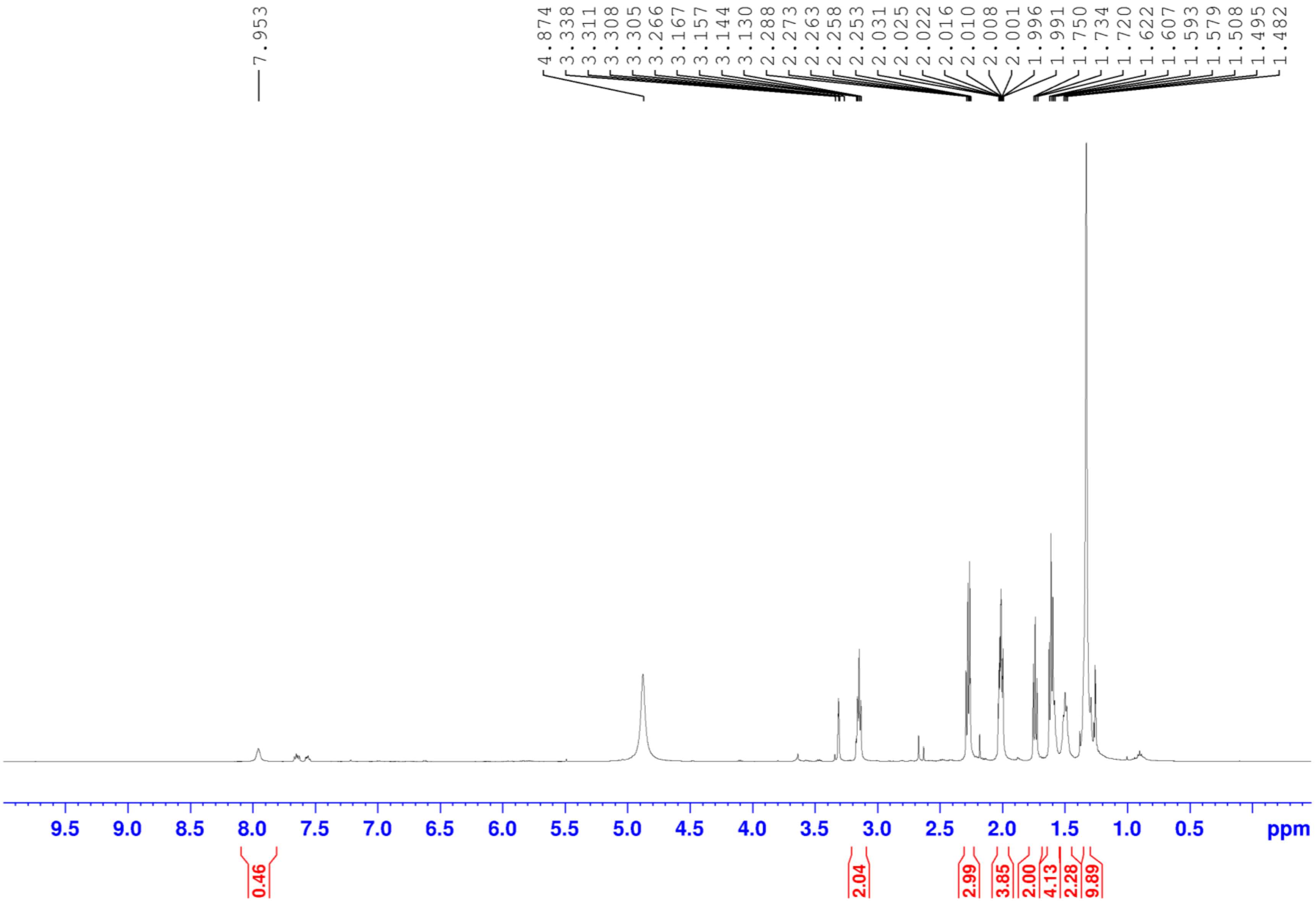

Compound **2** (**x-Alk-FA**) ^{13}C NMR

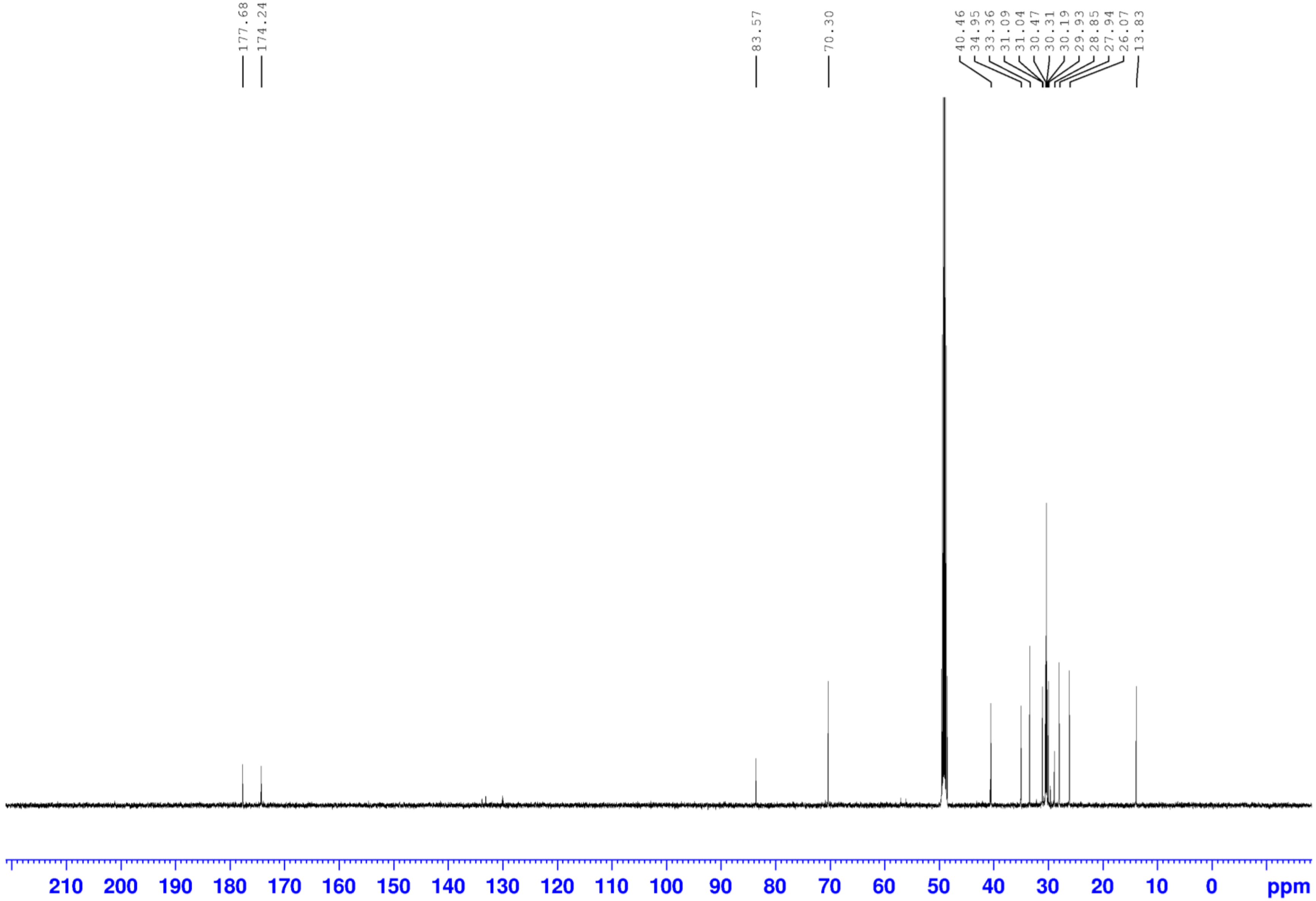

Compound **2** (**x-Alk-FA**) COSY NMR

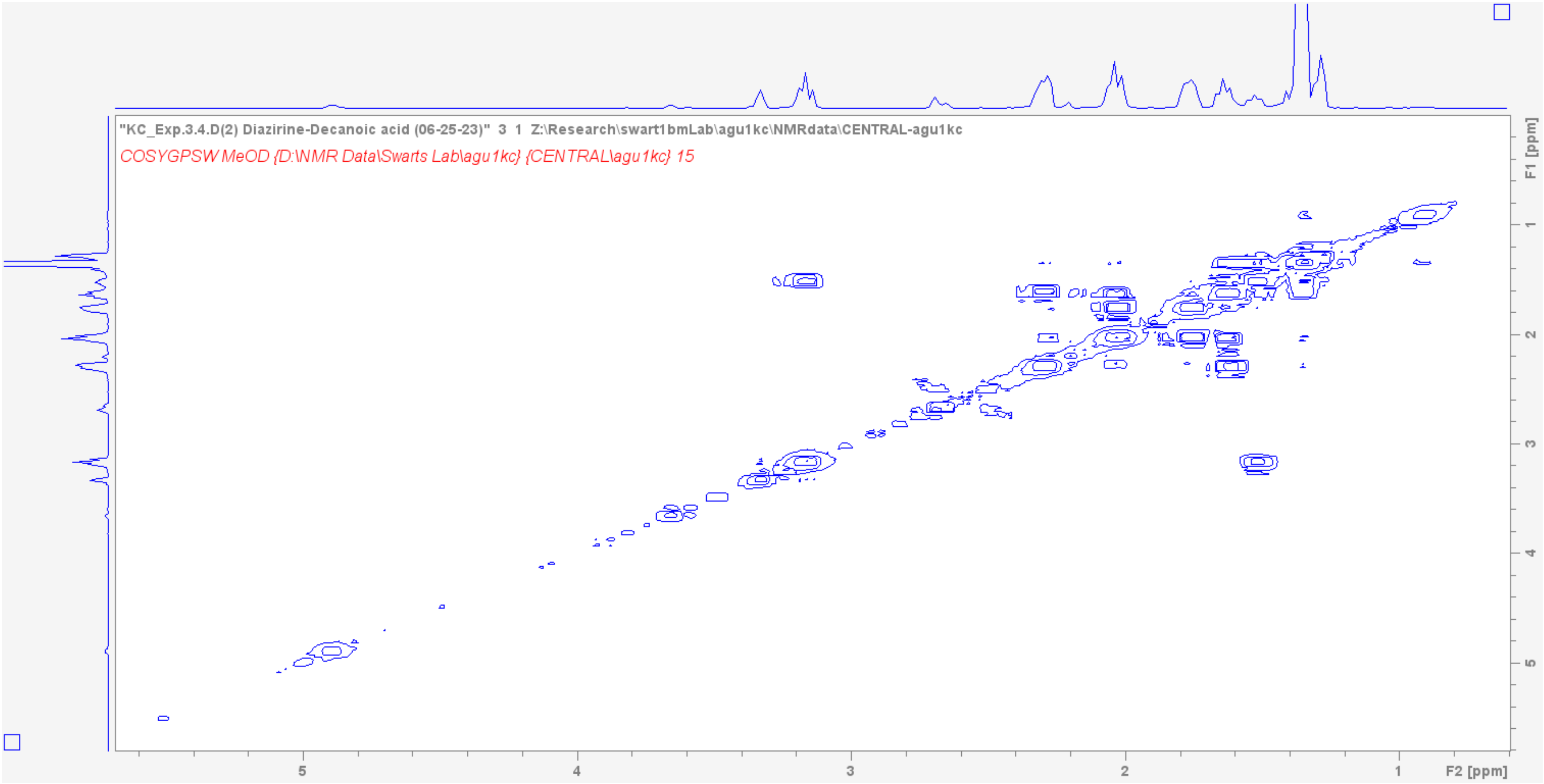

Compound **2** (**x-Alk-FA**) ESI MS

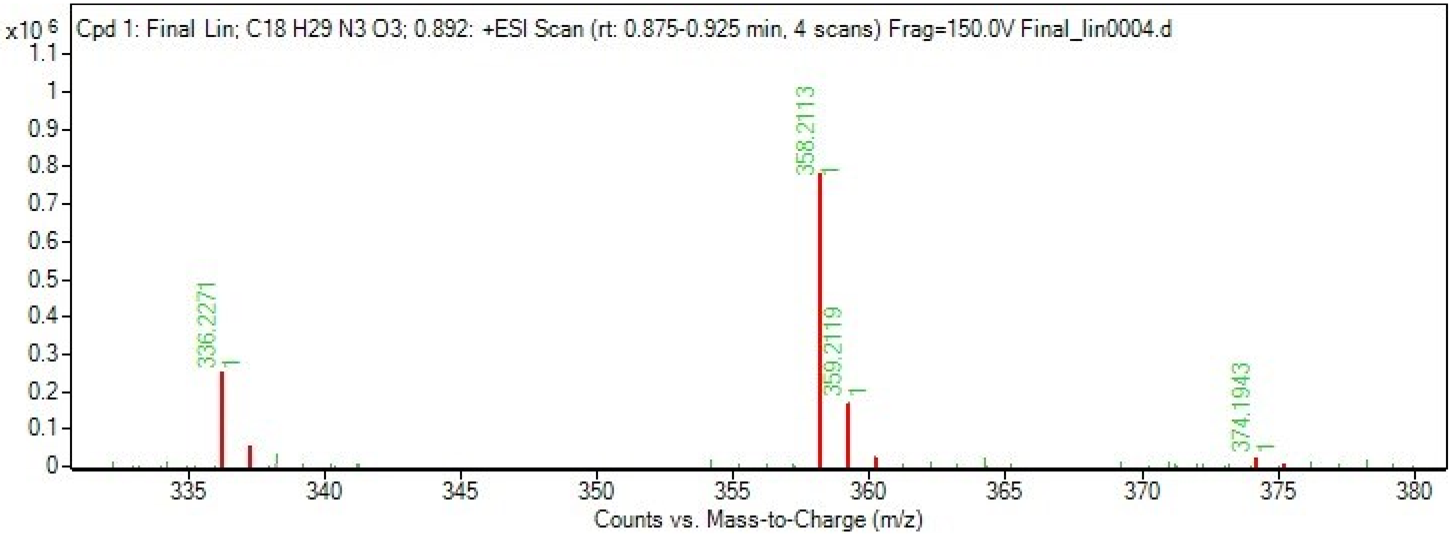

Supplement: Supplementary file 1 — id5c00068_si_001.pdf [file id5c00068_si_001.pdf]
